# Semi‐polar root exudates in natural grassland communities

**DOI:** 10.1002/ece3.5043

**Published:** 2019-04-29

**Authors:** Sophie Dietz, Katharina Herz, Stefanie Döll, Sylvia Haider, Ute Jandt, Helge Bruelheide, Dierk Scheel

**Affiliations:** ^1^ Department of Stress and Developmental Biology Leibniz Institute of Plant Biochemistry Halle (Saale) Germany; ^2^ Institute of Biology/Geobotany and Botanical Garden Martin Luther University Halle‐Wittenberg Halle (Saale) Germany; ^3^ German Centre for Integrative Biodiversity Research (iDiv) Halle‐Jena‐Leipzig Leipzig Germany

**Keywords:** exudates, grassland community, liquid chromatography coupled to mass spectrometry, plant functional traits, semi‐polar metabolites, untargeted metabolite profiling

## Abstract

In the rhizosphere, plants are exposed to a multitude of different biotic and abiotic factors, to which they respond by exuding a wide range of secondary root metabolites. So far, it has been unknown to which degree root exudate composition is species‐specific and is affected by land use, the local impact and local neighborhood under field conditions. In this study, root exudates of 10 common grassland species were analyzed, each five of forbs and grasses, in the German Biodiversity Exploratories using a combined phytometer and untargeted liquid chromatography‐mass spectrometry (LC‐MS) approach. Redundancy analysis and hierarchical clustering revealed a large set of semi‐polar metabolites common to all species in addition to species‐specific metabolites. Chemical richness and exudate composition revealed that forbs, such as *Plantago lanceolata* and *Galium* species, exuded more species‐specific metabolites than grasses. Grasses instead were primarily affected by environmental conditions. In both forbs and grasses, plant functional traits had only a minor impact on plant root exudation patterns. Overall, our results demonstrate the feasibility of obtaining and untargeted profiling of semi‐polar metabolites under field condition and allow a deeper view in the exudation of plants in a natural grassland community.

## 1 INTRODUCTION

The roots of terrestrial plants are embedded in a belowground “black box” surrounded by soil and a multitude of living organisms. This small habitat, the rhizosphere, is characterized by complex chemical, biological, and ecological processes (Bais, Weir, Perry, Gilroy, & Vivanco, 2006). The chemical interactions are mediated by among others low‐molecular‐weight metabolites (Dakora & Phillips, 2002; Faure, Vereecke, & Leveau, 2008) released by the plant root and thus called plant root exudates. Those consist of polar, semi‐polar, and a‐polar metabolites. The semi‐polar metabolites, which in most cases are products of the plant secondary metabolism, are highly diverse and involved in different kinds of interactions with the rhizosphere habitat. They are a part of the plant stress responses to abiotic factors such as temperature, light, and soil conditions (Badri & Vivanco, 2009; Lambers, Martinoia, & Renton, 2015; Lambers, Raven, Shaver, & Smith, 2008) but also fertilizer treatments (Vogt, 2010). In case of land use, however, the involvement is not fully understood. Herz et al. (2017) observed an increased root volume and lower root carbon‐to‐nitrogen ratio in case of higher land use. So, they postulated that this is linked to high resource acquisition of fast growing grassland species. However, the link between exudation of a plant root and land use intensity has not been found so far (Herz et al., 2018).

With regard to biotic relations, the release of semi‐polar metabolites mediates a multitude of interactions with the microbial soil communities (Bardgett & van der Putten, 2014; Lambers, Mougel, Jaillard, & Hinsinger, 2009), and also with neighboring plants (Bais, Park, Weir, Callaway, & Vivanco, 2004; Bais et al., 2006; Broeckling, Broz, Bergelson, Manter, & Vivanco, 2008; van Dam & Bouwmeester, 2016; Weir, Park, & Vivanco, 2004). Exuded phytotoxins reduce the establishment, growth, or survival of susceptible neighbors by altering respiration, membrane transport, germination, or shoot and root growth (Bais et al., 2006). *Arabidopsis thaliana* seedlings for instance have a reduced root length of primary root but an increased number of lateral roots when exposed to exudates of other plant species (Biedrzycki, Jilany, Dudley, & Bais, 2010). Also hogweed (*Heracleum mantegazzianum*) root exudates inhibit the germination and growth of other plant species (Jandova, Dostal, Cajthaml, & Kamenik, 2015). Most of these abiotic and biotic factors, however, have been related to plants in “one plant – one factor” experiments under controlled conditions (van Dam & Bouwmeester, 2016; Strehmel, Bottcher, Schmidt, & Scheel, 2014) or in ecological field experiments without the investigation of belowground exudation (Herz et al., 2017; Ravenek et al., 2014, 2016).

Similarly, the impact of plant's characteristics such as species’ identity, developmental stage (Aulakh, Wassmann, Bueno, Kreuzwieser, & Rennenberg, 2001; Chaparro et al., 2013), or plant functional traits (Aulakh et al., 2001; Herz et al., 2018, 2017) on the exudation pattern or the plants relation to its habitat, respectively, was performed under controlled conditions. Plant functional traits are thereby “characteristics, or trait values, at tissue‐to‐organismal scales that reflect their evolutionary history and mold their performance” (Reich & Cornelissen, 2014). In case of belowground traits, this includes root length, root volume, root respiration, nutrient uptake kinetics, root tissue nutrient content, and also the release of exudates (Bardgett, Mommer, & Vries, 2014). Making use of such traits is a very attractive approach as it would allow to generalize patterns observed across different species, and thus, bringing them together in a comprehensive framework. In a preceding study (Herz et al., 2018), the relation between polar root exuded metabolites and plant traits was investigated under natural conditions. The authors demonstrated that the exudate pattern was correlated with the root weight of the phytometers (Herz et al., 2018). However, these results only refer to a limited number of exuded metabolites. So far, it is unknown whether such correlations also hold true for the much more diverse semi‐polar secondary compounds released by a plant root (Dixon, 2001; Monchgesang et al., 2016).

The listed gaps in the knowledge of root exuded semi‐polar metabolites and their relationships to the environment are mainly due to the challenge in collecting root exudates in the field. Techniques as the collection of exudates in hydroponic or rhizobox systems are not applicable in field experiments. They are either collected under artificial growth conditions and, thus, have only limited relevance for soil conditions, or the technique is too complicated for a field approach (Oburger et al., 2013). Another often limiting point is the frequent use of targeted metabolic profiling. Most studies of exudates use targeted analyses of a predefined set of selected compounds, such as the phytoalexin camalexin (Millet et al., 2010), or compound classes, for example, coumarins (Fourcroy et al., 2014) and strigolactones (Kohlen et al., 2011). Although this approach allows studying the role of a particular metabolite in the rhizosphere network, it cannot reflect the complexity of the functional linkage of metabolites such as semi‐polar compounds in a complete exudate profile. An untargeted metabolomics approach, instead, allows the detection and, to some extent, identification of metabolites which otherwise would be neglected (van Dam & Bouwmeester, 2016; Peters et al., 2018).

Whereas the study of Herz et al. (2018) demonstrated that that polar metabolites can be analyzed under field condition, the present study tested whether the collection, sample preparation, and untargeted metabolite profiling method is applicable for semi‐polar metabolites under field conditions. Ten species of two different growth forms (forb and grass) were planted as phytometers in 54 existing grassland communities in the regions of the Biodiversity Exploratories in Germany (Fischer et al., 2010) to distinguish between species‐specific and environmentally induced exudate patterns. Their root exudate profiles were investigated for their composition, specific compounds, and correlation with biotic and abiotic influences. Considering the characteristics of semi‐polar metabolites, we hypothesized that (i) there are differences in metabolite composition between the growth forms. We further hypothesized (ii) that these differences are mainly driven by species specificity in the exudate profiles. The significant species‐specific compounds were further analyzed for a putative chemical metabolite classification. We further postulate that (iii) semi‐polar metabolite composition is influenced by biotic factors, here the local neighborhood of the plant community, and by abiotic factors, such as the locational impact (Plot), and land use intensity (LUI). Finally, (iv) we tested whether the correlations observed between 18 different above‐ and belowground plant functional traits and polar root exuded metabolites (Herz et al., 2018) also hold for semi‐polar metabolites compositions of the phytometer exudates.

## 2 MATERIAL AND METHODS

### 2.1 Experimental setup

The experiment was performed during March and September 2014 in the three regions of the German Biodiversity Exploratories (Fischer et al., 2010): Schorfheide‐Chorin, Hainich‐Dün, and Swabian Alb as described in Herz et al. (2017). In total, 18 experimental grassland plots in each exploratory were selected varying in land use intensity, leading to a total number of 54 plots. In total, 10 species were investigated in this experiment: five forbs (*Achillea millefolium* L. [Asteraceae], *Galium mollugo* L., *Galium verum* L. [Rubiaceae], *Plantago lanceolata* L. [Plantaginaceae], and *Ranunculus acris* L. [Ranunculaceae]) and five grasses (*Alopecurus pratensis* L., *Arrhenatherum elatius* [L.] P.Beauv. ex J.Presl & C.Presl., *Dactylis glomerata* L., *Lolium perenne* L., and *Poa pratensis* L. [all Poaceae]). These perennial species are among the most frequent and abundant species in all Exploratory grassland plots. Seeds of all species were collected in the Exploratories and raised under greenhouse conditions. Each species was planted into five subplots established in each of the 54 plots. Within a subplot, the plants were planted at a distance of 50 cm to each other at random locations. A full description of the field design and the planting procedure is given in Herz et al. (2017). Due to restricted access to some plots at the time of sampling and mortality in some plots, it could not be sampled the full set of planted phytometers. In total, 389 plants (*A. millefolium*: 38, *G. mollugo*: 41, *G. verum*: 37, *P. lanceolata*: 39, *R. acris*: 28, *A. pratensis*: 40, *A. elatius*: 40, *D. glomerata*: 48, *L. perenne*: 37, *P. pratensis*: 41) of 46 of the 54 experimental plots were sampled using the same approach described in Herz et al. (2018).

### 2.2 Collection of exudates

The procedure developed by Aulakh et al. (2001) was adapted for a field targeted analysis of semi‐polar metabolites. The phytometer plants were excavated after being exposed to field conditions for 3 months and the roots carefully washed with tap water to remove bulk and rhizosphere soil. After the reduction of possible ions from the tap water by a second wash step with deionized water, the complete roots of the intact plant were placed in 250 ml brown plastic vessels containing 200 ml of deionized water of HPLC quality for 2 hr exudation. To distinguish exudates from procedure artefacts, 200 ml water samples without exudation (“blank”) were treated exactly like the exudate samples. All samples were frozen and stored at −20°C until further processing. The samples were filtered after a slowly thawing process and reduced to soluble substances by evaporating the water under reduced pressure at 40°C in a rotary evaporator. The metabolites were resolved by dissolving them two times with 100% methanol (Sigma‐Aldrich, Taufkirchen, Germany), sonicating them for 10 min at 20°C, and then transferring the solution into new tubes. Followed by a second evaporation step (under pressure, 40°C) in a vacuum centrifuge, the metabolites were reconstituted in 80% methanol containing 20 µg/ml 2,4‐dichlorophenoxyacetic acid and 10 µM Ribitol as internal standards. An aliquot of 100 µl of exudate was transferred into a glass vial (Waters, Eschborn, Germany) and subjected to mass spectrometry. Further details, and additional tests on the appropriateness of the approach are given in Herz et al. (2018).

### 2.3 LC‐MS analysis and data processing

Exudates and controls were measured by nontargeted metabolite profiling with ultra performance liquid chromatography coupled to electron spray ionization quadrupole time‐of‐flight mass spectrometry (UPLC/ESI‐Q‐ToF‐MS). Performance was supervised by measurements of a standard mix of eight substances (MM8: 10 µM α‐Phenylglycin, 10 µM Kinetin, 10 µM Rutin, 10 µM o‐Anisic acid, 10 µM Phlorizin, 10 µM IAA Valine, 10 µM Indolacetonitril, and 10 µM Biochanin) every 10 samples.

The separation of metabolites was performed by ultra performance liquid chromatography (ACQUITY UPLC; Waters) equipped with a C18 column (ACQUITY UPLC HSS T3 Column, 100 Å, 1.8 µm, 1 mm × 100 mm; Waters) with 2 µl full loop injection at 40°C. The following gradient was utilized: flow rate of 150 µl/min 0–1 min, isocratic 95% A (water/formic acid, 99.9/0.1 (v/v)), 5% B (acetonitrile/formic acid, 99.9/0.1 (v/v)); 1–14 min, linear from 5% to 95% B; 14–18 min, isocratic 95% B; 18–20 min, isocratic 5% B. The eluting compounds were detected from m/z 90 to 1,000 using a MicrOTOF–Q II hybrid quadrupole time‐of‐flight mass spectrometer equipped with an Apollo II electrospray ion source (Bruker Daltonics, Billerica, MA, USA) in negative ion mode. The following instrument settings were used: nebulizer gas, nitrogen, 1.6 bar; dry gas, nitrogen, 6 L/min, 190°C; capillary, −4,000 V; end plate offset, −500 V; funnel 1 RF, −200 Vpp; funnel 2 RF, −200 Vpp; in‐source CID energy, 0 eV; hexapole RF, −100 Vpp; quadrupole ion energy, −5 eV; collision gas, nitrogen; collision energy, −7 eV; collision −150 Vpp; transfer time, 70 µs; prepulse storage, 5 µs; spectra rate, 3 Hz.

The individual raw data files were recalibrated on lithium formate cluster ions obtained by automatic infusion of 20 µl 10 mM lithium hydroxide in isopropanol/water/formic acid, 49.9/ 49.9/0.2 (v/v/v) at a gradient time of 18 min and by using a diverter valve. Peak picking was performed with Compass DataAnalysis 4.4.2 software (Bruker Daltonics) and a signal‐to‐noise threshold of 2, correlation coefficient threshold 0.7, minimum feature length 7 spectra, smoothing width of 3. Automated detection of adducts was enabled for M‐H, M‐H_2_O‐H, M+Na‐H2, M+K‐H2 and M + HCHOOH‐H. No background subtraction was performed. Retention time alignment and feature extraction was performed by Compass ProfileAnalysis 2.3 (Bruker Daltonics) within the retention time (RT) range of 0.01–18.00 min and mass range of 90–1,000 mass to charge ratio (m/z). Peak grouping was performed with an allowed deviation of 0.1 min for the retention time and 200 mDa for the mass. Features occurring only once in all samples were excluded from further analysis. An automated annotation of the feature list by m/z and RT was done with MetaboScape 2.0 (Bruker Daltonics). Features occurring in 50% of water controls were excluded from the metabolite list. The feature list was subjected to statistical analysis (see below).

### 2.4 LC‐MS/MS analysis and data processing

For the acquisition of collision‐induced dissociation (CID) mass spectra, exudate samples and water controls were pooled according to their affiliation to the 10 species and three Exploratory sites. The measurements were performed by UPLC/ESI‐Q‐ToF‐MS with an ultra performance chromatographic system (ACQUITY UPLC; Waters) equipped with a C18 column (ACQUITY UPLC HSS T3 Column, 100 Å, 1.8 µm, 3 mm × 100 mm; Waters) and a MicrOTOF–Q I hybrid quadrupole time‐of‐flight mass spectrometer equipped with an Apollo II electrospray ion source (Bruker Daltonics). The separation and MS measurement were performed as described above. CID mass spectra were acquired at first by automated data dependent MS/MS and, if necessary, using a scheduled precursor ion list with an isolation width of ±3–15 m/z and fragmentation inside the collision cell with an applied collision energy in the range of 15–70 eV. Argon was used as collision gas. Product ions were detected using the same parameter settings as described above. MS as well as tandem MS measurements of pooled samples were processed with MetaboScape 3.0.1 software (Bruker Daltonics). The T‐Rex 3D algorithm (Bruker Daltonics) was applied for peak picking, alignment, and automated assignment of MS2 spectra with following settings: intensity threshold: 1,500, minimum peak length: seven spectra, minimum peak length recursive: seven spectra, minimum of compounds for extraction: 2, no log mass calibration, primary ion: M‐H‐, expected ions: M+Cl‐, M+Na‐H‐, M+K‐H‐, ions for pseudo spectra: M‐H‐H2O, M+HCOOH‐H‐, EIC correlation; 0.8, mass range: 90–1,001, RT range: 0.01–18.

### 2.5 Identification approach

In a first step, significant species‐specific compounds were annotated according their m/z and RT manually. Secondly, the most probable elemental composition was calculated with MetaboScape 3.1’s Smart Formula algorithm (Bruker Daltonics). In a third step, MS/MS spectra of compounds (limited to the species‐specific metabolites) were exported and the spectral library of species‐specific compounds was processed with the MetFamily metabolite classification online software (Treutler et al., 2016). There fragment spectra were deconvoluted, reduced to fragments with an intensity above 1,000. Afterward, fragment intensities were normalized within each MS/MS spectrum to a maximum of 1 (base peak) (Treutler et al., 2016). Fragments with a normalized intensity higher 0.1 and neutral losses were annotated according to their m/z similarity to those of an in‐house database of possible characteristic fragments and neutral losses measured on LC‐MS systems (Supporting Information Appendix S1: Table A1). Using this database and further chemical knowledge, a putative classification of compounds was performed. The results were summarized in Supporting Information Appendix S1: Table A2.

### 2.6 Plant functional trait analysis and ecological data

After sampling exudates, the plant material was used for analyzing plant functional traits (Supporting Information Appendix S1: Table A3) as described in detail in Herz et al. (2017). Fresh and dry mass of roots were assessed, shoots and leaves separately. Roots and leaves were scanned fresh on a HP Scanjet Flatbed Scanner at 600 dpi and analyzed with the programs WinRHIZO (v Pro 2008a; Regent Instruments, Quebec, Canada) and WinFOLIA (v Pro 2004a; Regent Instruments). From these measurements, root volume, root mass per volume, and specific leaf area (SLA) (leaf area per total dry weight) were obtained. We are aware that root mass per volume differs from root tissue density measurements made by diameter class (Rose, 2017). Given the large amount of samples that had to be processed, it was not possible to analyze all roots systems by diameter class. However, comparisons between plants are not affected, as all samples were treated in the same way. Additionally, the root and leaf dry matter content (RDMC, LDMC) (root and leaf dry mass per fresh mass, respectively) as well as root to shoot ratio (RSR) was calculated. The dried roots were ground to assess C, N (C/N‐analyzer vario EL cube; Elementar, Hanau, Germany) and after a digestion with nitric acid also P (photometric phosphate assay), K, Mg, and Ca (atom absorption spectrometry with AAS vario 6; Analytik Jena, Jena, Germany) content as well as C to N ratio.

The composition of the local neighborhood of the phytometers was obtained by recording the number of plant species and cover per species growing in a 15 cm radius (707 cm^2^) around each phytometer plant. These records were used to calculate species richness and Shannon diversity of the local neighborhood, in addition to species composition as obtained from the first four axes of a detrended correspondence analysis (DCA).

### 2.7 Statistical analysis

All statistical analyses were performed with R (version 3.4.4; R Core Team, 2015). All analyses were carried out on exudates based on a presence/absence matrix of these compounds since compound number instead of intensity was in focus of this study, similar to the analysis of Herz et al. (2018). To calculate the chemical richness of each species, the mean of number of measured metabolites per group was calculated. This was used as base for calculation of the overall significance of the difference between species by an analysis of variance (ANOVA) (function aov; R Core Team, 2015). A Scheffé post hoc test (function scheffe.test, package agricolae; de Mendiburu, 2017) was performed to test for the exact significant differences between groups and presented in a violin plot (function ggplot and geom_violin, package ggplot2; Wickham, 2009). To investigate the exudate composition of the 10 species, a hierarchical clustering (function dist and hclust; R Core Team, 2015) was performed. Moreover, a redundancy analysis (RDA) (function rda, package vegan; Oksanen et al., 2016) was conducted to relate the matrix of semi‐polar metabolites to the presence/absence matrix of the species from which the exudates were obtained.

To calculate the number of shared compounds between all species, all compounds that occurred at least twice in all analyzed samples were taken into consideration and summed up per species. The species specificity of compounds was analyzed by calculating the frequency of all compounds per species and using performing an exact binomial test (function binom.test; R Core Team, 2015). We tested the probability of every compound to be more frequent in a particular species than in the other nine species together (*p* > 0.95). These species‐specific compounds were further used for the tandem MS approach and the putative identification approach. To test for the relationship between above‐ and belowground traits and exudation patterns of semi‐polar exudates matrix of 302 of the 389 samples of each traits (Supporting Information Appendix S1: Table A3) and exudates was subjected to a Principal Component Analysis (PCA) (function rda, package vegan; Oksanen et al., 2016) and then compared both matrices with a Procrustes analyses (function protest, package vegan; Oksanen et al., 2016). The trait matrix was thereby rotated to reach maximum similarity with the exudate matrix. The Procrustes rotation minimizes the sum of squared differences between both matrices. Then, a permutation test is used to obtain the correlation between both matrices. Furthermore, the data were subjected to variance partitioning (function varpart, package vegan; Oksanen et al., 2016) to detect how much variance was explained by either the combination of target species identity (Species), the environment and geographic location (Plot), composition of the local neighborhood of each phytometer (LNH) or the combination of Species, Plot and plant functional traits of the phytometer (Trait). Variance partitioning was performed separately for the two growth forms to reveal the differences in variation between grasses and forbs. Furthermore, the explained variance for each single variable of the predictors Trait, LNH, and LUI (Blüthgen et al., 2012) was calculated, together with Species and Plot using variance partitioning (function varpart, package vegan; Oksanen et al., 2016). Here, LUI is the mean of land use intensity index of the years 2006–2014. Each LUI consists of the sums of measurements of each plot (*i*) of fertilization (*F*, in kg nitrogen ha^−1^ year^−1^), the frequency of mowing (per year), and grazing intensity which were standardized relative to their means (*r*) within the sites (Blüthgen et al., 2012).

$$\text{LUI}=\frac{F_{i}}{F_{r}}+\frac{M_{i}}{M_{r}}+\frac{G_{i}}{G_{r}}.$$

## 3 RESULTS

### 3.1 Profiles of semi‐polar metabolites differ more due to the species identity than due to growth form

**Figure 1:**
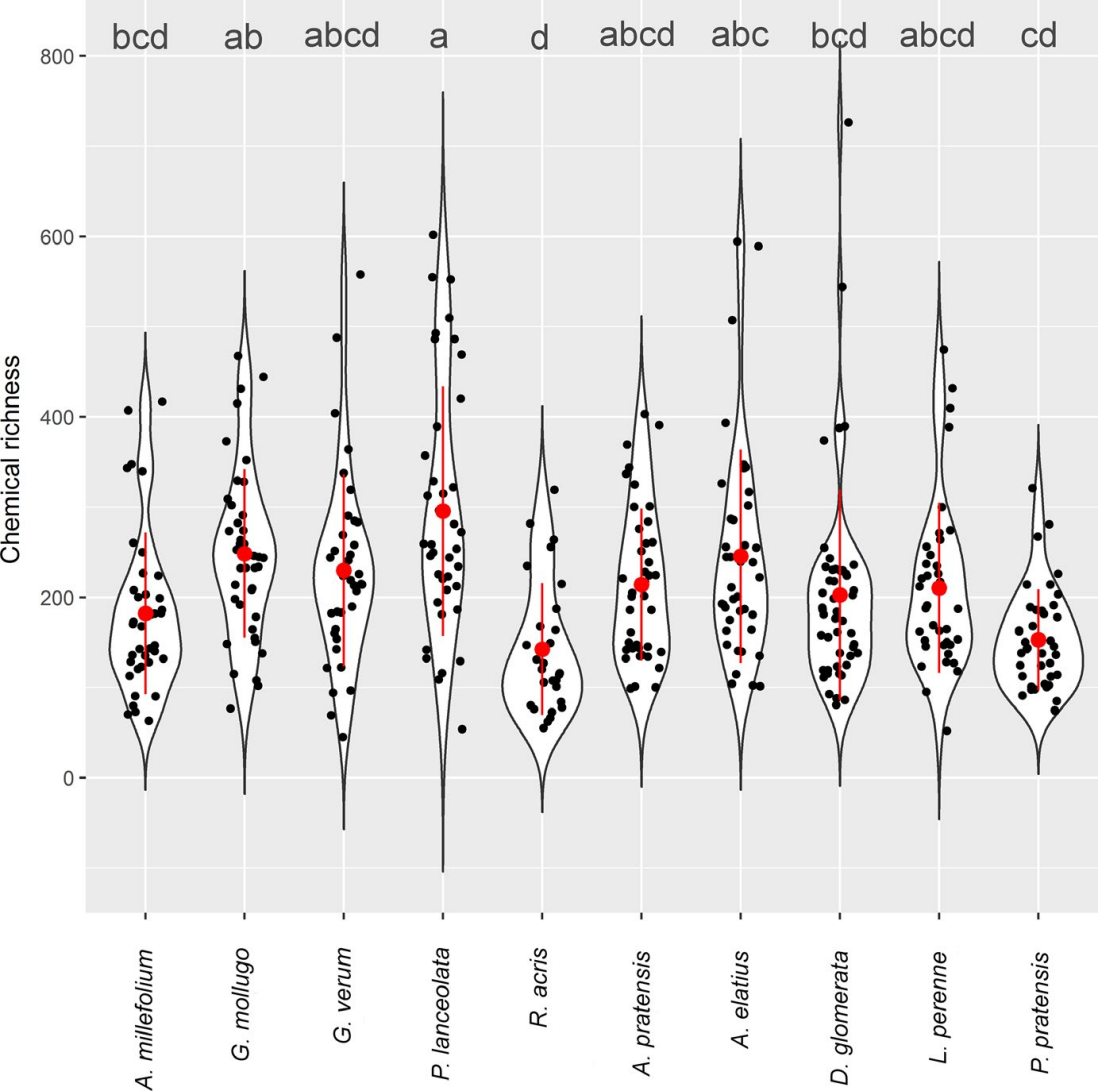
Chemical richness of semi‐polar metabolites in root exudates of 10 grassland species. Violin plot presents the number of measured compounds per species (chemical richness) of the 389 exudate samples. The shape of the violins represents the distribution of the number of metabolites. The black points show the value of the specific samples. Red points represent the median of the chemical richness, whereas the lines represent the quantiles. ANOVA with the median of chemical richness as response and species as predictor revealed a significant influence of species with a *p*‐value of 2.42e‐10***. The Scheffé Post hoc test uncovered significant differences between the species, presented by letters. Violins with the same letters are not significantly different from each other

**Figure 2:**
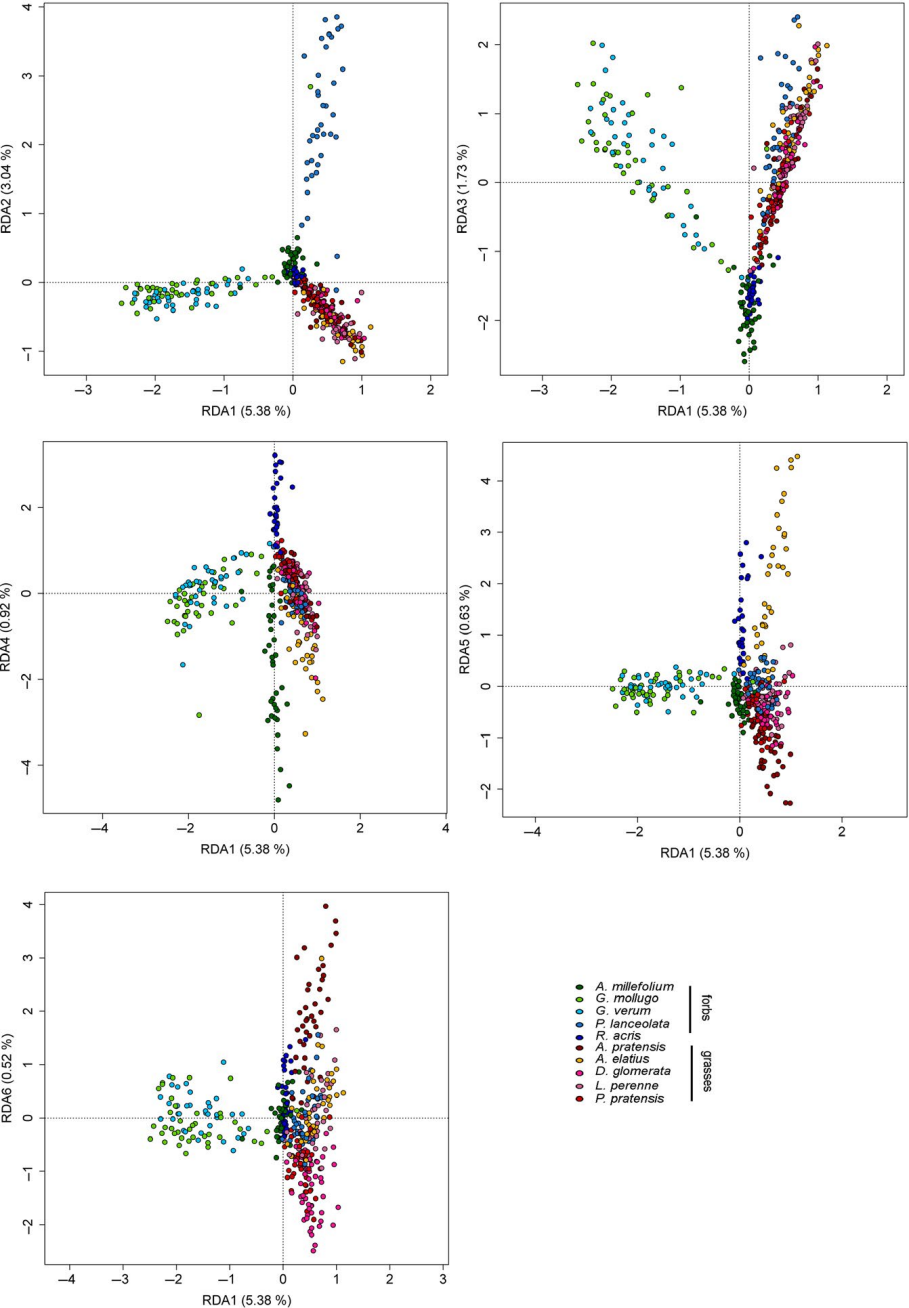
Redundancy analysis of semi‐polar metabolites in root exudates. RDA was performed with 389 samples plotted against a presence/absence matrix of species. Axes one to six are displayed. The 10 species are represented by color (see legend). RDAs colored by growth form are presented in Supporting Information Appendix S1: Figure A1

**Figure 3:**
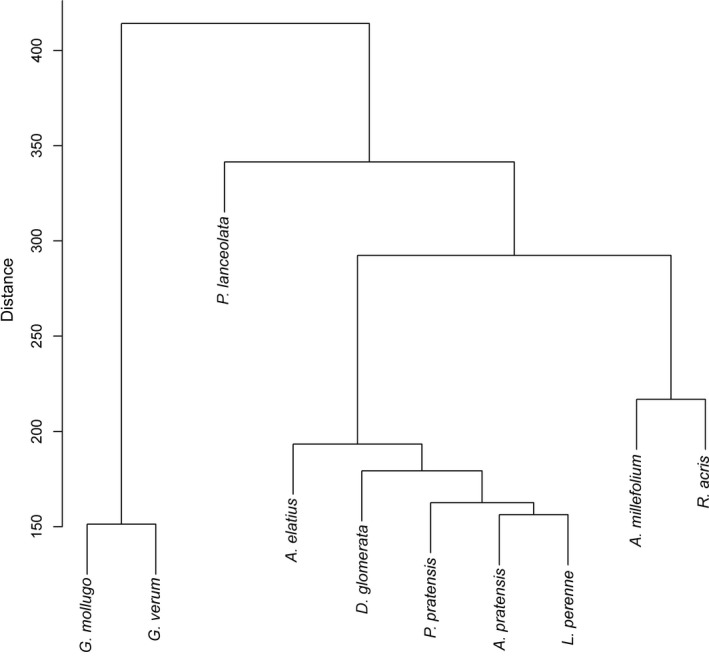
Hierarchical clustering of phytometer sample according to their semi‐polar exudate composition. The 389 samples were clustered according to their differences in the semi‐polar metabolite composition by a distance‐based analysis of all annotated compounds

Using the untargeted metabolite profiling approach, 5,414 features were annotated as putative compounds among the 389 phytometer exudate samples. An ANOVA of the chemical richness of each species revealed that the total exuded number of compounds of each species differs significantly between species (*p* < 0.001, Figure 1), but not between the two growth forms (*p* = 0.0684). Thus, *P. lanceolata *displays a significantly higher chemical richness than *A. millefolium*, *D. glomerata*, *P. pratensis,* and *R. acris*, whereas *G. mollugo* has a significantly higher chemical richness than *P. pratensis* and *R. acris *but not higher than *P. lanceolata*. Moreover, *R. acris* showed a significant lower chemical richness than *A. elatius*. All other species did not differ significantly in the number of exuded metabolites. This trend was also observed in a redundancy analysis (RDA) (Supporting Information Appendix S1: Figure A1) where separation of grass exudation profiles was less pronounced than that of forbs. There the samples of the species *G. mollugo *and *G. verum* cluster together on the first axis (5.38%), whereas the separation of *P. lanceolata* occurred on the second axis (3.04%) apart from the *Galium* species and the other species (Figure 2). A further separation of *A. millefolium* and *R. acris* was observed on axis three (1.72%), axis four (0.92%), and less prominent on axis five (0.63%) (Figure 2). The exudate profiles of two of the grass species were separated at first on the axis five (*A. elatius*), and more or less on the axis six (0.52%, *A. pratensis* as well as a cluster of *D. glomerata, L. perenne* and *P. pratensis*). Distance‐based hierarchical clustering of exudate patterns resulted in a similar picture (Figure 3) indicating that the exudation profiles are species‐dependent. It also points to more similar exudation patterns among grasses than among forbs.

### 3.2 Species‐specific semi‐polar metabolites

The analysis of samples concerning shared and unique semi‐polar metabolites revealed that 270 compounds occurred in both growth forms, whereas 625 compounds were significantly species‐specific. Specific metabolites were more pronounced in forbs (534) than in grasses (91). In addition, 150 of exuded compounds were specific for the two species of the *Galium* genus (*Galium* spp.). Specific metabolites were more pronounced in forbs (534) than in grasses (91). In addition, 150 of exuded compounds were specific for the two species of the *Galium* genus (*Galium* spp.). Following our identification approach, 200 of these compounds were chosen for further identity elucidation (Supporting Information Appendix S1: Table A2). A total of 102 of these compounds could be assigned to the following metabolite families (Table 1, Supporting Information Appendix S1: Table A2): glycosides (26; one sulfated glycoside, one sulfated and phosphorylated glycoside, one diglycoside), phenylpropanoids (64) with one glycosylated coumarin (1), polyphenols, such as flavonoids (23; 12 of them glycosylated), other polyphenols (10; 8 of them are hydroxycinnamic acids and one of them is a glycosylated hydroxycinnamic acid), as well as other not further sub‐classified phenylpropanoids (29; 11 of them are glycosylated). Furthermore, compounds of the classes polyketides (3) and terpenes (6; two were glycosylated) were found, as well as three iridoid glycosides. Furthermore, a total of 104 unclassified compounds with different functional groups were also detected. Many of the compound classes occurred in different species at the same time. Glycosides were mainly detected in *A. millefolium* and *P. lanceolata*, but occurred also in *G. mollugo* as well as in both *Galium* spp. together and one time in *R. acris* and *A. elatius*. Flavonoids occurred mainly in *Galium* spp. and specific ones in *G. mollugo* and to a lower extent in *G. verum*, but also in *P. lanceolata*, *A. elatius* und *P. pratensis*. Glycosylated flavonoids were predominantly observed in exudate samples of *Galium* spp. and *P. lanceolata*. Also hydroxycinnamic acid and glycosylated phenylpropanoids were mainly detected in *Galium *spp. and *P. lanceolata*, whereas unglycosylated phenylpropanoids mainly occurred in *P. lanceolata*. Glycosylated terpenes could only be annotated in *P. lanceolata* and *A. elatius *samples.

**Table 1:** Putative classification of species‐specific compounds

			*A. millefolium*	*G. mollugo*	*G. verum*	*Galium spp*.	*P. lanceolata*	*R. acris*	*A. pratensis*	*A. elatius*	*D. glomerata*	*L. perenne*	*P. pratensis*
Glycoside (26)		Glycoside (23)	8	2		3	8	1		1			
		Glycoside, sulfated (1)					1						
		Glycoside, sulfated, phosphorylated (1)					1						
		Diglycoside (1)								1			
Phenylpropanoid (64)		Coumarin, glycosilated (1)				1							
	F. (23)	Flavonoid (11)		2	1	5	1			1			1
		Flavonoid, glycosilated (12)	1	1		3	6						1
	Other Polyphenols (10)	Other Polyphenol (1)				1							
		Hydroxy‐cinnamic acid (8)	1			2	3		1	1			
		Hydroxy‐cinnamic acid, glycosylated (1)					1						
	Other P. (29)	Phenylpropanoid (18)^a^	4			2 (1)^a^	9	1				2	
		Phenylpropanoid, glycosylated (11)	1			4	4	1		1			
		Polyketide, aromatic acetate (3)		1	1	1							
		Jasmonate conjugate (2)					2						
Terpene (6)		Terpene (1)								1			
		Terpene, glycosylated (2)					1			1			
		Iridio glycoside (3)					3						
Unclassified (104)		Unclassified (73)	12	6		25	18	11			1		
		Unclassified, aromatic acid fragment (5)					4			1			
		Unclassified, Imine (2)				2							
		Unclassified, phosphorylated (3)				1	1	1					
		Unclassified, phosphorylated, glycosylated (1)								1			
		Unclassified, sulfated (11)	1			1	6	2		1			
		Unclassified, glycosylated (5)					2	2		1			
		Unclassified, sulfated, phosphorylated (1)					1						
		Unclassified, sulfated, glycosylated (3)					2	1					

The table contains the total number of compounds (in brackets) putatively classified as one of the respective metabolite classes as well as the occurrences in the samples of the ten different species.

a Fragment spectrum of compound contains characteristic ion, which could also account for Agmatine classification.

Beside shared classes, there were also some of them exclusively exuded by one out of 10 species (Table 1, Supporting Information Appendix S1: Table A2). The three putatively classified iridoid glycosides (931.2832 m/z, 4.03 min; 667.15112 m/z, 4.04 min; 585.16223 m/z, 4.84 min) and two putative jasmonate‐related metabolites (661.29873 m/z, 5.19 min; 499.23636 m/z, 5.88 min; 485.2021 m/z, 5.79 min), as well as the sulfated (701.23201 m/z, 4.84 min) and sulfated and phosphorylated glycosides (591.22857 m/z, 4.42 min), exclusively occurred in *P. lanceolata *samples. Also, a glycosylated hydroxycinnamic acid (431.1292 m/z, 3.45 min) was detected only in *P. lanceolata *samples. The polyketides only occurred in the *Galium* spp. samples. *A. elatius* was the only species in which one putative terpene (563.2187 m/z, 5.92 min) and a diglycoside (975.5086 m/z, 6.76 min) were detected.

### 3.3 Biotic and abiotic impact on exudate pattern based on growth form

**Figure 4:**
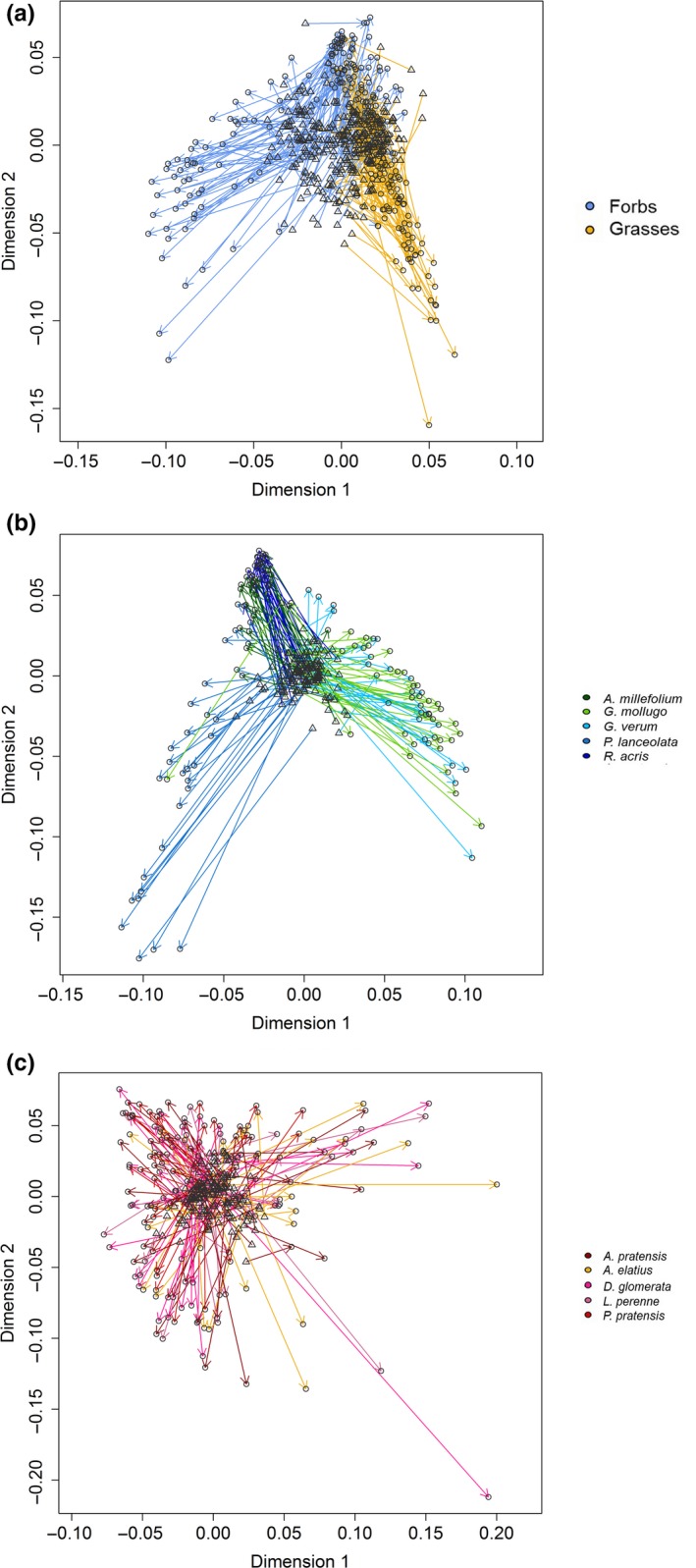
Procrustes analysis of Principal Component Analysis (PCA) of plant functional traits and PCA of semi‐polar metabolites in root exudates. PCAs of Supporting Information Appendix S1: Figures A4 and A5 were correlated to each other. Direction of stretch of the ordination of plant functional trait composition (triangles) to the ordination of the exuded semi‐polar metabolite composition (circles) is shown by arrows. (a) Procrustes analysis colored by growth form (see legend), Correlation of the symmetric Procrustes rotation = 0.4582, *p* = 0.001, Number of permutations = 999. (b) Procrustes plot of the forb samples colored by species (see legend) Correlation of the symmetric Procrustes rotation = 0.1919, *p* = 0.001, Number of permutations = 999 (c) Procrustes plot of the grass samples colored by species (see legend). Correlation of the symmetric Procrustes rotation = 0.2592, *p* = 0.001, Number of permutations = 999

**Figure 5:**
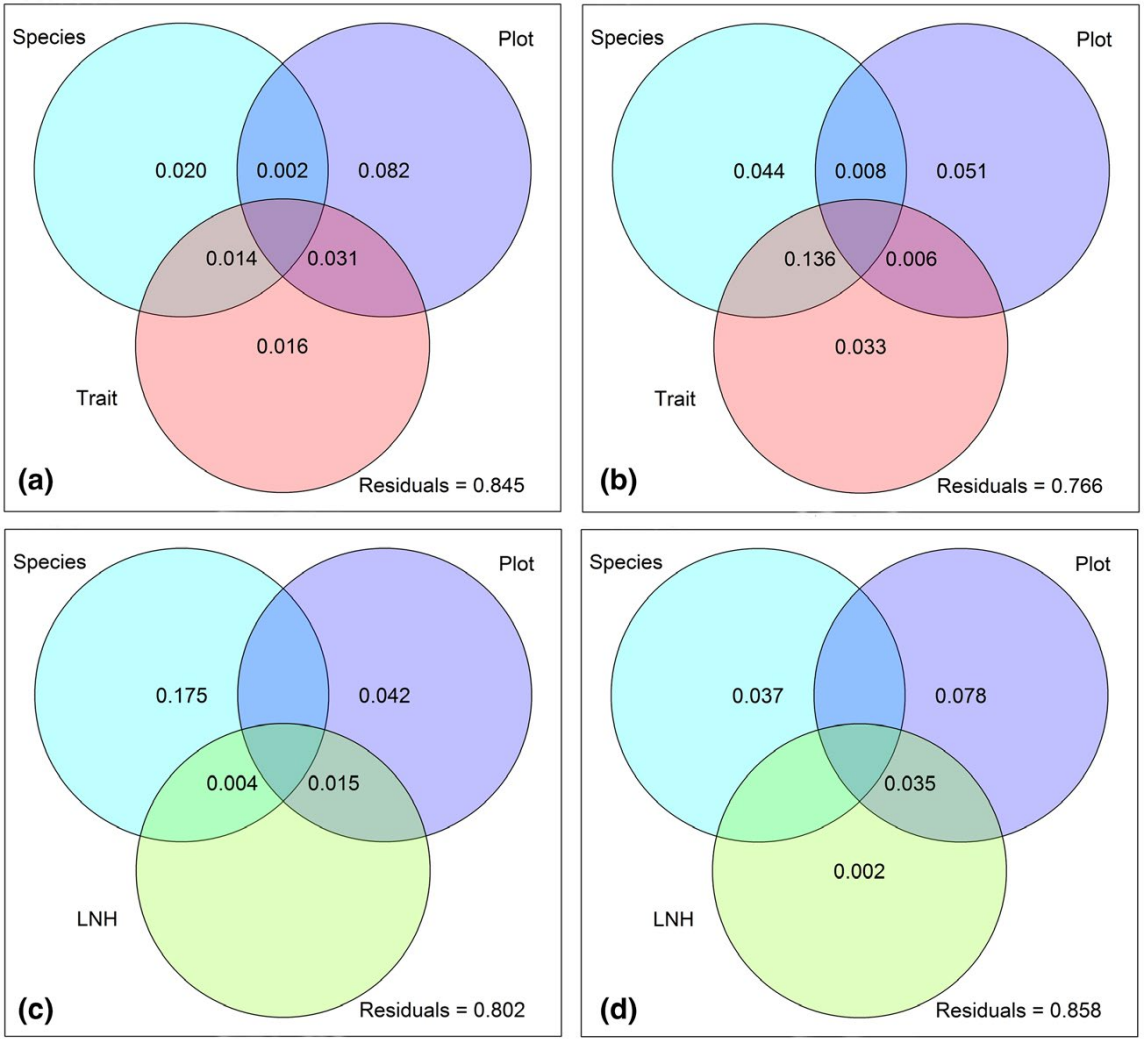
Variance partitioning for the composition of semi‐polar metabolites in root exudates. Graphs represent the proportion of explained variance in the semi‐polar metabolites composition of forbs (a, c) and grasses (b, d). Predictor variables: Species = species identity of the phytometer; Plot = impact of environment and geographical location of the plot; LNH = species composition of the local neighborhood (containing variables determined in 15 cm radius around the phytometer, including species richness, Shannon diversity, total cover and species composition of the local neighborhood, Traits = plant functional traits presented in Supporting Information Appendix S1: Table A3. Values below 0 are not shown

Many biotic and abiotic factors have an impact on the exudation profiles of plants (van Dam & Bouwmeester, 2016; Eisenhauer et al., 2017). However, it is unknown what kind of factors affect semi‐polar metabolite exudate pattern of plants under field conditions. Therefore, correlational analyses of semi‐polar root exudates as well as ecological conditions and plant functional traits were measured on 302 of the same 389 phytometers (Supporting Information Appendix S1: Figure A2). A Procrustes correlation (Figure 4) of a Principal Component Analysis (PCA) of exuded semi‐polar metabolites (Supporting Information Appendix S1: Figures A3, A5, A6) and a PCA of plant functional traits (Supporting Information Appendix S1: Figures A4, A7, A8) indicated a connection between plant functional traits and exudation patterns of semi‐polar metabolites (*R* ^2^ = 0.79, *p* = 0.001). The variance partitioning of exudate composition with the predictors’ species identity (Species), the locational impact (Plot), and above‐ and belowground plant functional traits (Traits) (Figure 5, Supporting Information Appendix S1: Table A3) could only partly explain these results. Traits explained less of the variance in exudate pattern of both growth forms (forbs: 3.3% and grasses: 1.6%) than the other predictors. In forbs (Figure 5a), Plot explained 5.1% of variance and Species 4.4%, whereas a large proportion of variance in exudate composition was simultaneously related to Species and Traits (13.6%). In Grasses (Figure 5b), Plot explained the exudate composition variance best (8.2%), followed by Species (2.0%), whereas Plot and Traits together explained only a minor part of variance (3.1%). This overall observation did not change when plant local neighborhood community (LNH) was included. Instead, LNH accounted for none of the variance in exudate composition in forbs (0%, Figure 5c) and for only a small proportion of variance in grasses (0.2%, Figure 5d). The ranking of predictors did not change in grasses (Plot: 7.8%, Species: 3.7%, Plot and LNH: 3.5%, Figure 5d), whereas in forbs (Figure 5c) the predictor Species had the highest explanatory power (17.5%) followed by Plot (4.2%) and simultaneously explained variance by Plot and LNH (1.5%). A marginal shared explained variance occurred due to Species and LNH (0.4%). A more detailed analysis of the individual explained variances of the contributing variables to the predictors Traits and LNH (Table 2) revealed that in case of forbs dry mass of roots and leaves (1.5% each), carbon (C) concentration of roots (1.5%) and specific leaf area (1.0%) contributed each to a minor extent to the total explained variance of the Traits. In case of grasses, C concentration of roots explained 1.4% of variance whereas the proportion of explained variance given by dry mass of leaves and roots (2.0% each) was higher. The contributing variables for predictor LNH and LUI, as an indirect contributing variable to predictor Plot, did not contribute to the explained variance at all (Table 2).

**Table 2:** Proportion of explained variance of single variables implemented in the predictors of the variance partitioning of semi‐polar metabolite composition of root exudates in both growth forms

(a) Forbs									
Predictor	single Variable (SV)	Species	Plot	SV	Species + Plot	Plot + SV	Species + SV	Species + Plot + SV	Residu‐als
Plant functional traits	SLA	14.56	5.47	1.03	−1.89	0.21	3.4	−1.71	78.93
	LDMC	16.17	5.7	0.04	−2.43	−0.02	1.78	−1.17	79.93
	LAR	17.96	5.59	−0.07	−3.61	0.09	−0.01	0.01	80.03
	RSR	18.07	5.79	0.25	−3.58	−0.1	−0.11	−0.02	79.71
	RDMC	17.69	5.73	0.11	−3.6	−0.05	0.26	−0.01	79.85
	RMV	17.79	5.71	0.19	−3.49	−0.03	0.16	−0.12	79.78
	RVol	13.97	6.12	0.43	−1.08	−0.43	3.98	−2.52	79.53
	RCC	16.79	6.33	1.54	−3.07	−0.65	1.16	−0.53	78.42
	RNC	17.56	5.68	0.24	−3.22	0	0.39	−0.38	79.72
	RCNR	17.3	5.49	−0.07	−3.21	0.2	0.65	−0.39	80.03
	RPC	10.59	5.61	0.07	−1.39	0.07	7.37	−2.21	79.89
	RKC	17.99	5.54	−0.11	−3.61	0.15	−0.03	0	80.08
	RMgC	17.54	4.58	−0.14	−3.16	1.11	0.42	−0.44	80.1
	RCaC	17.66	5.07	−0.08	−3.4	0.62	0.29	−0.2	80.04
	DM_leaves	15.27	5.63	1.49	−2.06	0.05	2.69	−1.54	78.47
	DM_roots	15.37	5.6	1.52	−2.25	0.09	2.58	−1.35	78.44
	DM_total	14.96	5.45	0.5	−2.11	0.24	2.99	−1.49	79.47
	DM_above	15.66	5.45	0.48	−2.49	0.24	2.29	−1.11	79.48
	LUI	17.95	5.49	0	−3.47	0.19	0	−0.14	79.96
Plant local neighborhood community (LNH)	Cover	17.99	5.54	−0.11	−3.61	0.15	−0.03	0	80.08
	DCA1	17.54	4.58	−0.14	−3.16	1.11	0.42	−0.44	80.1
	DCA2	17.66	5.07	−0.08	−3.4	0.62	0.29	−0.2	80.04
	DCA3	17.91	5.55	−0.09	−3.55	0.14	0.04	−0.05	80.05
	DCA4	17.58	5.66	0.08	−3.23	0.02	0.38	−0.38	79.89
	Richness	18.13	5.61	−0.05	−3.68	0.08	−0.18	0.07	80.01
	Shannon	18.2	5.6	0.01	−3.69	0.09	−0.25	0.09	79.95

(b) Grasses									
Predictor	single Variable (SV)	Species	Plot	SV	Species + Plot	Plot + SV	Trait + SV	Species + Plot + SV	Residu‐ales
Plant functional traits	SLA	3.33	10.78	1.93	−0.77	0.58	0.14	−0.14	84.15
	LDMC	3.28	11.2	−0.06	−0.69	0.16	0.2	−0.22	86.14
	LAR	3.46	11.12	−0.05	−0.75	0.24	0.01	−0.15	86.13
	RSR	3.41	11.4	0.08	−0.89	−0.05	0.06	−0.02	86
	RDMC	3.29	11.3	0.11	−0.8	0.06	0.18	−0.11	85.97
	RMV	3.43	11.33	−0.01	−0.87	0.02	0.04	−0.03	86.09
	RVol	3.32	11.39	0.04	−0.83	−0.04	0.15	−0.07	86.04
	RCC	3.38	11.46	1.39	−0.77	−0.1	0.09	−0.13	84.69
	RNC	3.19	11.56	0.35	−0.79	−0.2	0.28	−0.12	85.73
	RCNR	3.3	10.17	0.08	−0.62	1.19	0.17	−0.28	85.99
	RPC	3.24	10.72	0.17	−0.67	0.63	0.23	−0.24	85.91
	RKC	3.48	11.3	0.07	−0.9	0.06	−0.01	0	86.01
	RMgC	3.52	9.55	−0.07	−0.91	1.8	−0.05	0	86.15
	RCaC	3.52	10.42	−0.01	−0.92	0.93	−0.05	0.01	86.08
	DM_leaves	3.16	10.72	1.98	−0.69	0.64	0.31	−0.21	84.1
	DM_roots	3.15	10.76	2.01	−0.68	0.59	0.32	−0.22	84.07
	DM_total	3.34	10.72	0.83	−0.77	0.64	0.13	−0.13	85.25
	DM_above	3.34	10.65	0.62	−0.77	0.71	0.13	−0.14	85.46
	LUI	3.47	10.92	0	−0.92	0.43	0	0.02	86.08
Plant local neighborhood community (LNH)	Cover	3.48	11.3	0.07	−0.9	0.06	−0.01	0	86.01
	DCA1	3.52	9.55	−0.07	−0.91	1.8	−0.05	0	86.15
	DCA2	3.52	10.42	−0.01	−0.92	0.93	−0.05	0.01	86.08
	DCA3	3.52	10.86	0.05	−0.92	0.5	−0.05	0.01	86.03
	DCA4	3.48	11.27	0.09	−0.88	0.09	−0.01	−0.03	85.99
	Richness	3.52	10.5	−0.01	−0.82	0.86	−0.05	−0.09	86.09
	Shannon	3.48	10.44	0.02	−0.77	0.92	0	−0.13	86.06

The Explained variance (in %) is given for single variables for each predictor and both growth forms, a) forbs and b) grasses, separately. Negative amounts of explained variance are caused by unbalanced sample sizes and can be considered to be 0.

Cover: cover of all vascular plant species in a 15‐cm radius around each phytometer; DCA1‐4: first four axes of a detrended correspondence analysis of all plants surrounding each phytometer; plant functional traits = see Table S3; LUI: Land use intensity index; Richness: species richness of local neighbourhood; Shannon: index for local neighbourhood diversity.

In conclusion, the variance partitioning of both predictor combinations revealed that forb exudates were more related to species identity and displayed a higher inter‐specific variation than those of grasses. Grass exudation patterns instead were more responsive to geographic and environmental impacts than forbs.

The variance partitioning also revealed that a higher total variation in root exudation was explained in case of forbs (27.8%–23.6%, Figure 5a,c) than in case of grasses (16.5%–15.2%, Figure 5b,d) although the proportion of unexplained variance was high in both growth forms (76.6%–80.2% in case of forbs, 84.5%–85.8% in case of grasses). This points to further influencing factors in addition to those analyzed in this study.

## 4 DISCUSSION

The analysis of plants' belowground biochemistry is challenging especially under field conditions (van Dam & Bouwmeester, 2016). For instance, the proximity of a target root to those of other plants makes it difficult to collect the roots of interest without damaging them. Therefore, the collection of exudates of plants is usually performed under hydroponic (Monchgesang et al., 2016) or field mimicking conditions (Eisenhauer et al., 2017; Petriacq et al., 2017). In contrast, the approach presented here and in Herz et al. (2018) allowed the sampling of root exudates from plants grown in soil in their natural habitat. It could be shown that the collection method caused only insignificant micro‐injuries of plant root tissues (see Herz et al., 2018). This was made possible by the application of an early stage phytometer approach, where the roots did not grow into other root networks (Clements & Goldsmith, 1924; Dietrich, Nilsson, & Jansson, 2013). This prevented root tissue injury due to intertwined roots during the sampling and allows an actual insight into the rhizosphere network. Thus, to our knowledge, this is one of the first investigations of the exudate composition of semi‐polar metabolites in a complex environmental context.

The applied statistical analysis of exudate composition of the 10 different species showed that the exuded semi‐polar metabolite patterns of forbs are different from those of grasses. However, the here presented results point to a higher inter‐specific variation in exudation patterns in forb than in grass species, since *P. lanceolata* and *Galium* species differ also from *A. millefolium *and *R. acris*. This partly contradicts the first hypothesis. At the same time, it supports the second hypothesis, since the differences between growth forms originate from specificity of particular species. Species specificity of root exudates has already been described in earlier studies (Badri & Vivanco, 2009; van Dam & Bouwmeester, 2016; Monchgesang et al., 2016).

The grass species, however, did not differ from each other to a large extent. On the one hand, that might be traced back to the closer phylogenetic relation between the chosen grasses (all are members of the *Poaceae* family) than those between the investigated forbs. On the other hand, it could also be that the responses of grasses are more similar to each other as they are the dominant life form, and thus, best reflect the ecological selection pressure present in grasslands. The higher similarity among grasses is in accordance with the result of the variance partitioning and published ecological trait studies (Aerts & Chapin, 1999; Craine, Froehle, Tilman, & Chapin, 2001; Freschet, Cornelissen, Logtestijn, & Aerts, 2010; Herz et al., 2017; Pérez‐Harguindeguy et al., 2013; Roumet, Urcelay, & Diaz, 2006; Siebenkas, Schumacher, & Roscher, 2015; Tjoelker, Craine, Wedin, Reich, & Tilman, 2005). For instance, Herz et al. (2017) and Siebenkas et al. (2015) investigated variation in belowground traits between forbs and grasses and found that grasses have a higher plasticity in their traits due to their better adjustment in this habitat. In accordance with this, forbs have to integrate much more into this habitat which fits the explained variance of Plot. The explained variance of grass exudate composition, however, was low for all predictors. This also points to further factors affecting exudate composition than the edaphic and climatic plot conditions included in our study.

The data presented here provide furthermore evidences that plant functional traits are linked to exudation of these plants, which confirms hypothesis iv. Biomass‐related traits, such as dry mass of roots and leaves or carbon concentration, contribute to explained variance of exudate profiles of both growth forms, grasses, and forbs. This is in accordance with the result of Aulakh et al. (2001) who observed that plant biomass alter the exudate pattern of rice plants. In future, investigation of the effects of the single functional traits on the composition of exudates and the occurrence of single exuded compounds could help to better understand their role in the process of exudation.

Interestingly, there was no evidence that the exudate composition of the phytometers is affected by the plant local neighborhood community, which contradicts our hypothesis iii and the literature (Biedrzycki et al., 2010; Cheng et al., 2007; Jandova et al., 2015; Vogt, 2010). One reason for this lack of neighborhood effect might be the short exposure time of the phytometers to the new habitat. The findings of Ravenek et al. (2014) point to such a possibility, since they observed changes in morphological belowground traits only after 4 years in their analysis of long‐term influences of biodiversity effects on belowground biomass. However, these findings were determined for root functional traits and not for exudates. Thus, the correlation between exudates and exposure time to a certain environment has to be investigated in follow‐up experiments with a longer field growing period.

It is also of great interest that there was no observed impact of LUI to the exudate pattern of the phytometers. This also contradicts the hypothesis iii and stands in contrast to other studies presenting an interaction between LUI (Blüthgen et al., 2012) and, for example, soil biota (Blüthgen et al., 2012) or plant traits (Herz et al., 2017) in the Biodiversity Exploratory (Fischer et al., 2010). The LUI represents a broad combination of fertilization, mowing, and livestock grazing at each site in one parameter. Unfortunately, it accounts only for an annual average of each parameter, whereas measurements for parameters at the exact sampling time, such events as trampling and number of fertilization per plant, are missing. This limitation could account for the lack of measurable influence.

Overall, the total amount of explained variation was unexpectedly low. This points to further factors influencing the exudation of plant roots. For instance, it could be possible that the semi‐polar exudates detected in this study are more responsible for communication and defense (Badri, Weir, Lelie, & Vivanco, 2009; Strehmel et al., 2014). This is supported by the classification results and published functions of semi‐polar (secondary) exuded metabolites. Phenylpropanoids such as coumarins are involved in the regulation of oxidative stress and hormonal regulation (Treutter, 2006). Flavonoids protect plants against phytopathogens (Bourgaud et al., 2006; Treutter, 2006) and are involved in the activation of nodulation genes in various rhizobia species (Long, 1989). They have also been described as chemoattractants for *Rhizobium leguminosarum *biovar *meliloti* (Caetano‐Anolles, Crist‐Estes, & Bauer, 1988). Hydroxycinnamic acids act as phytoalexins in soil and inhibit the growth of other plants, as described for the solanaceous crops, eggplant (*Solanum melongena*) and tomato (*Lycopersicum esculentum*) (Yoshihara, Takamatsu, & Sakamura, 1978). Terpenes and terpenoids are involved in the plant defense against herbivores. Rasmann et al. (2005) identified (E)‐β‐caryophyllene exuded by maize roots into the soil as a “herbivore‐induced underground signal that strongly attracts entomopathogenic nematodes.” Also iridoid glycosides and the two jasmonate‐related metabolites, which both were detected exclusively in *P. lanceolata *exudates in this study, function as signals in plant herbivore interactions (Rosenthal & Berenbaum, 2012; Schweiger, Heise, Persicke, & Muller, 2014). Furthermore, terpenoids act as phytoalexins against fungi and bacteria but are also used as energy source by bacteria (Langenheim, 1994). So far, it is unclear which role these metabolites fulfill in the field belowground network of the chosen phytometer. So, it would be of great interest to further investigate the identity of these metabolites by structure elucidating methods and their function by bioassays. An implementation of information of other abiotic, for example, soil and climate characteristics, and biotic factors, for instance microbial community, could further help to define their role in the belowground network in future experiments. However, the classification method of semi‐polar exuded compounds presented here is a substantial progress in untargeted profiling of metabolites and a good base for the investigation of the unknown compounds of such exudates.

The results on semi‐polar exudates contradict in some part those of Herz et al. (2018) on polar metabolites. Whereas LNH and LUI were of minor importance in both cases, the observed higher dependence of root exudation of polar metabolites to environmental impacts irrespective of the growth form (Herz et al., 2018) could not be confirmed in case of semi‐polar metabolites. This suggests that the different functions of these two types of metabolites depend differently on the environment. Although both kinds of metabolites are involved in the acquisition of nutrients and the attraction of beneficial interaction partners, semi‐polar metabolites are also released for the defense against harmful microorganisms, herbivores, and competing plants (Badri et al., 2009; Biedrzycki et al., 2010; van Dam, 2009; Jandova et al., 2015). Since this mechanisms evolved over time and in a species‐specific manner, the semi‐polar metabolite patterns are more diverse than those of polar (primary) metabolites (Dixon, 2001).

In conclusion, the results presented here provide information on the root exudate composition of plants exposed to a complex environment. Thereby, an unknown diversity and specificity of semi‐polar metabolites were demonstrated across these so far not inspected species. A novel method for classification of unknown compounds was employed, which otherwise would not have been classified. With this, the study provides a deeper insight in the exudation of forbs and grasses in a natural grassland community and demonstrates the feasibility of investigating semi‐polar metabolites in field studies.

## CONFLICT OF INTEREST

None declared.

## AUTHOR CONTRIBUTIONS

HB, UJ, SH and DS designed the field experiment. KH, SD, UJ and SH conducted the field experiment and collected the phytometers. Trait analysis was performed by KH. Exudate extraction and analyses were performed by SD. Annotation and identification were performed by SD with contribution by StD. Statistical analysis was performed by SD with input from HB, KH and DS. The manuscript was written by SD with input from all co‐authors.

## DATA AVAILABILITY

The mass spectrometric data are available from MetaboLights database (https://www.ebi.ac.uk/metabolights/MTBLS671) and www.bexis.uni-jena.de. The plant functional trait data are also available from www.bexis.uni-jena.de and TRY (https://www.try-db.org/TryWeb/Data.php, https://doi.org/10.17871/TRY.18). Information concerning land use intensity data that support the findings of this study is available from www.bexis.uni-jena.de.
