# Supplementary material for: Semi‐polar root exudates in natural grassland communities
Source: Ecol Evol. 2019 Apr 29;9(10):5526–41. doi: 10.1002/ece3.5043 (PMC6540716; doi:10.1002/ece3.5043)
Supplement: Supplementary file 1 [file ECE3-9-5526-s001.pdf]

Appendix

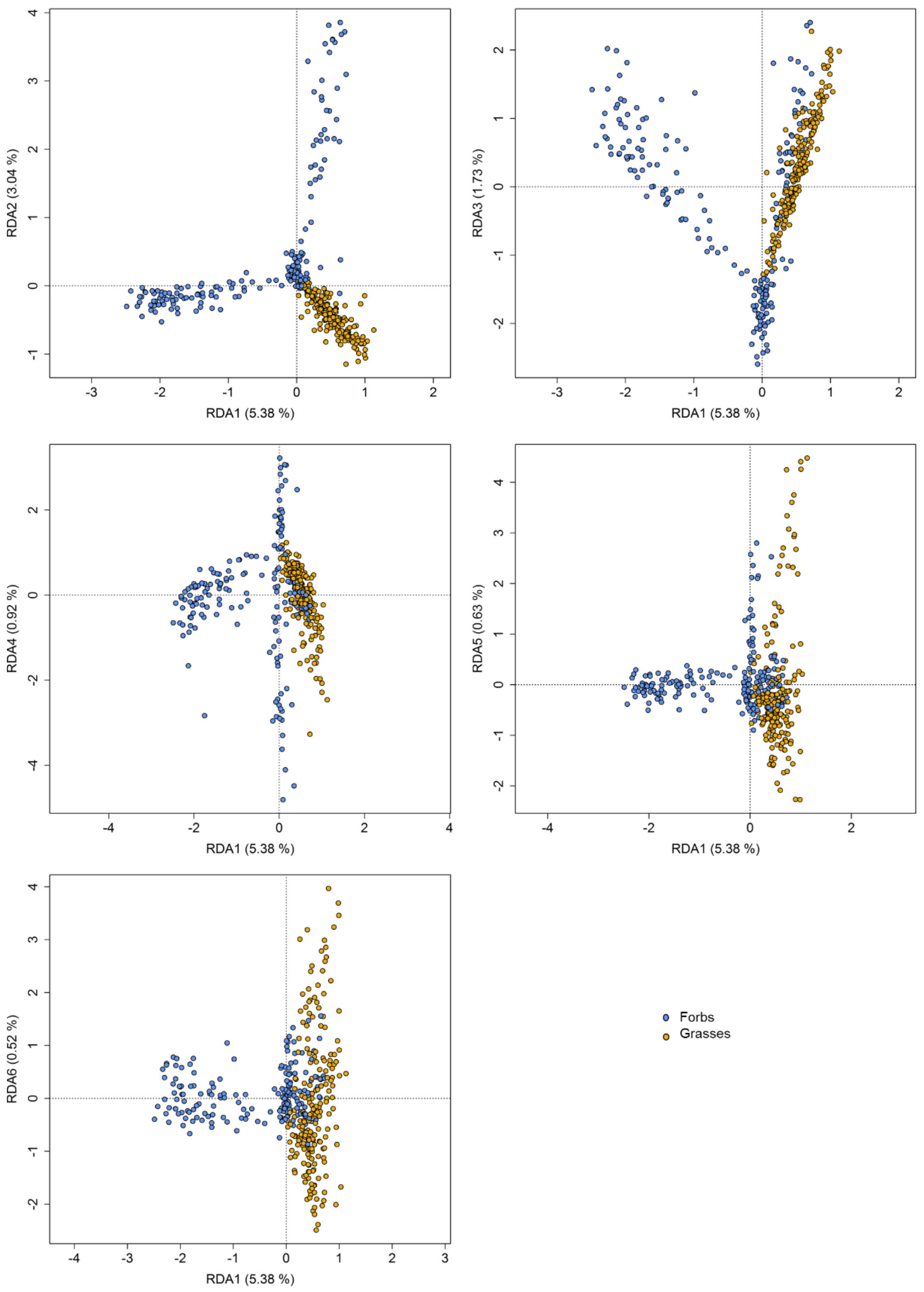

Figure A1: Redundancy analysis of semi-polar metabolites in root exudates. RDA was performed with 389 samples plotted against a presence/absence matrix of species. Axis one to six are displayed. The two growth forms are represented by colour (see legend).

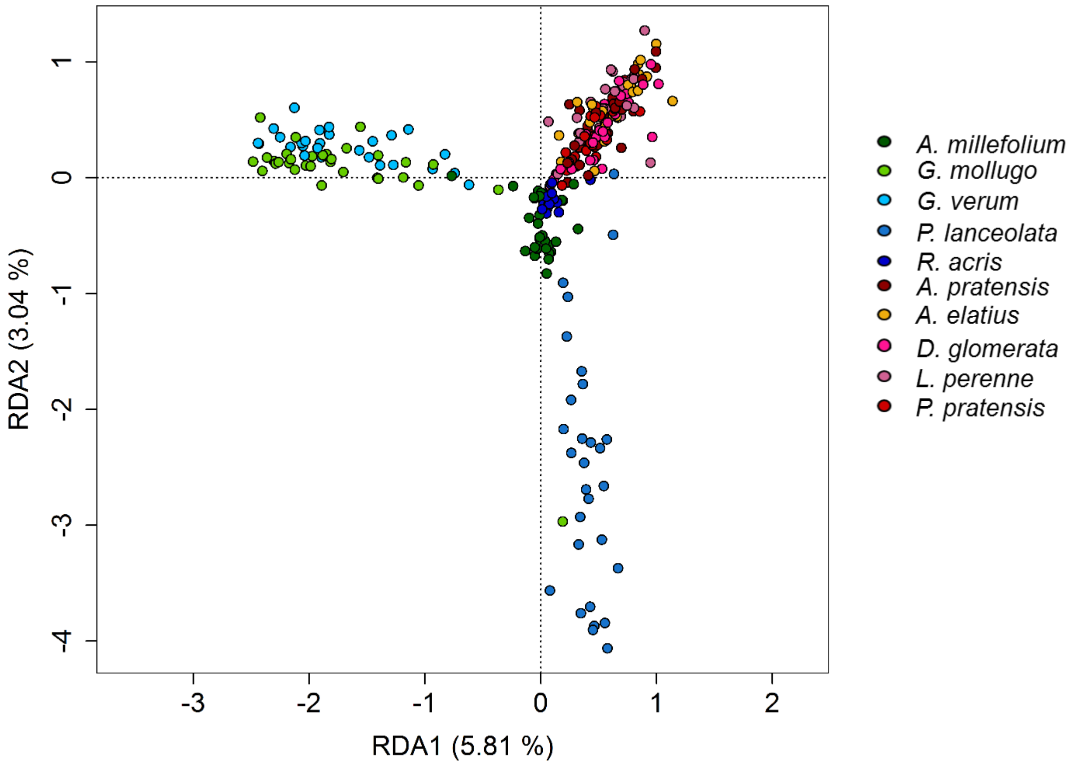

Figure A2: Redundancy analysis with semi-polar metabolites of root exudates. RDA was performed with the 302 samples for which also trait data were available. Metabolite compositions of the samples were plotted against a presence/absence matrix of species per site. The ten species are represented by colour (see legend).

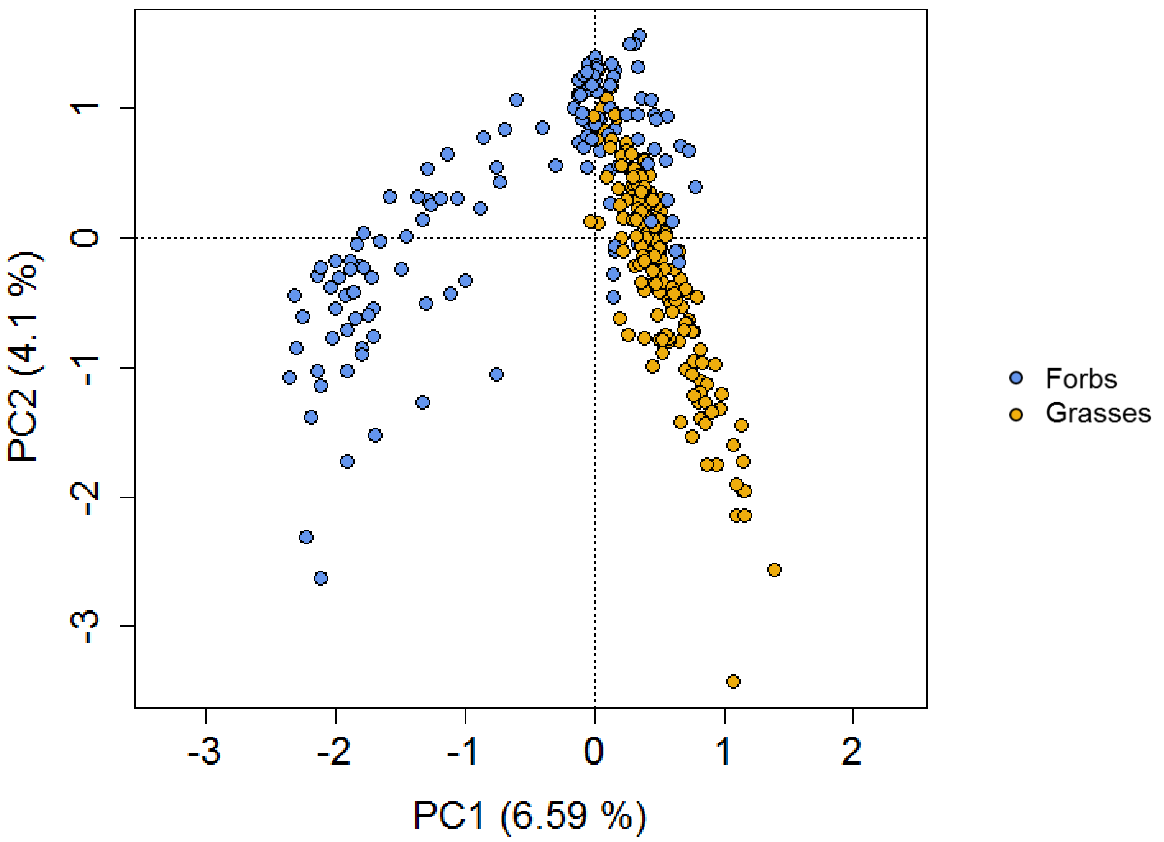

Figure A3: Principal component analysis of semi-polar metabolites in root exudates. PCA was performed with the 302 samples for which semi-polar metabolite data were also available. Colours represent growth forms (see legend).

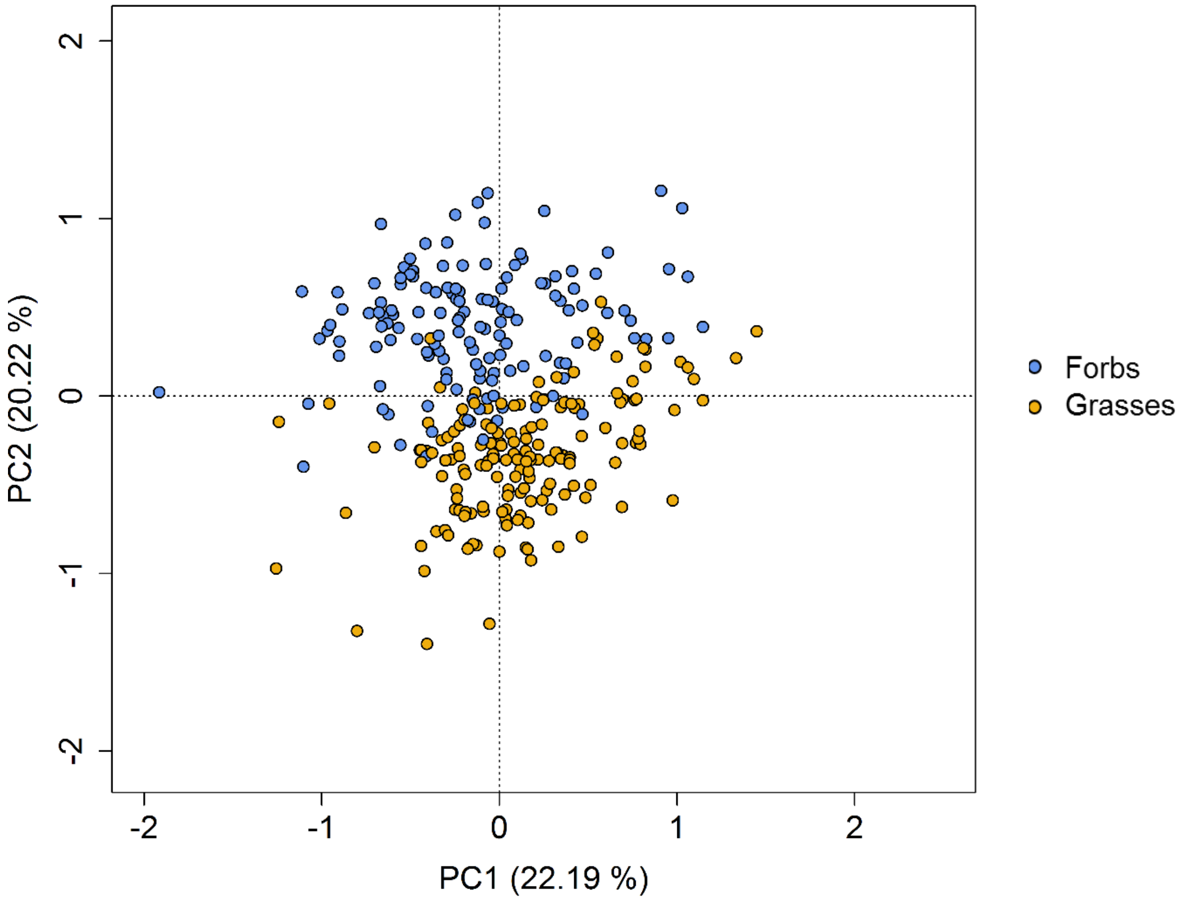

Figure A4: Principal component analysis of plant functional traits. PCA was performed with the 302 samples for which trait data were also available. Colours represent growth forms (see legend).

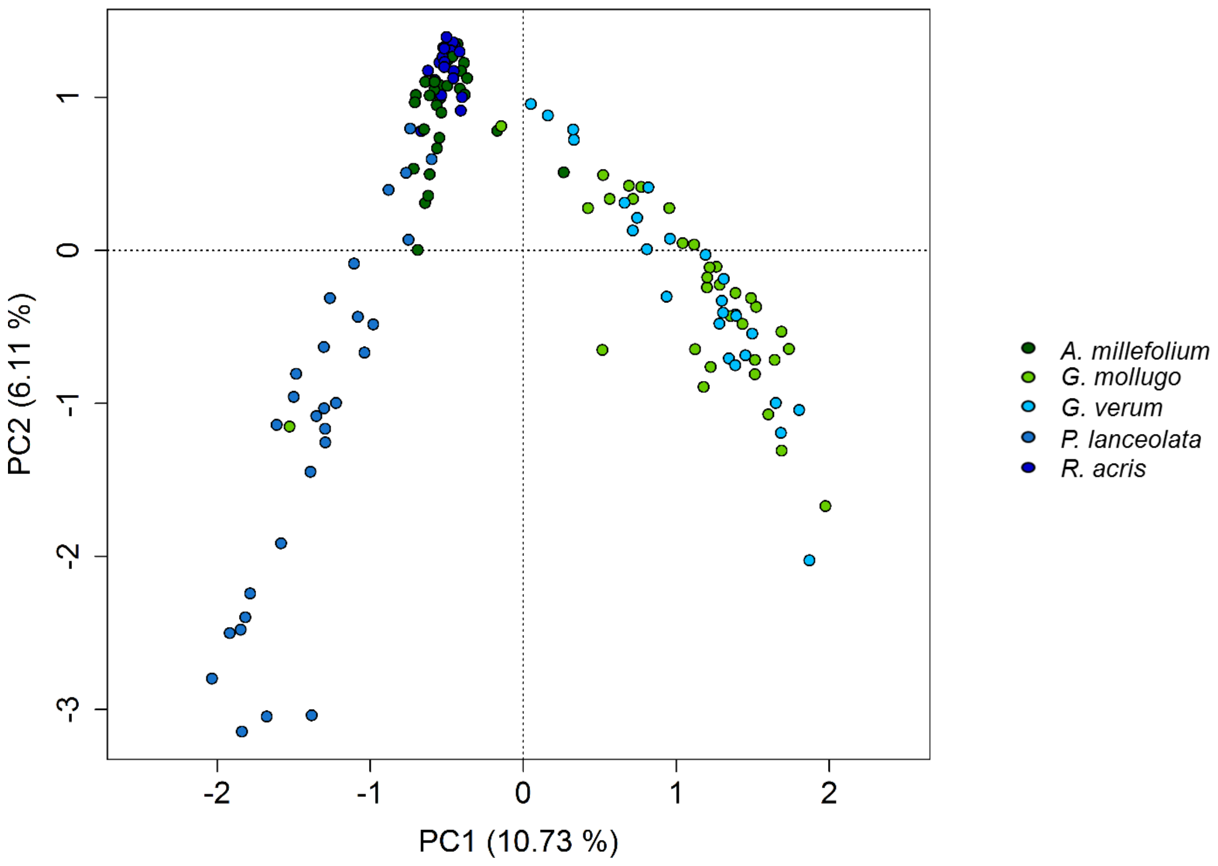

Figure A5: Principal component analysis of semi-polar metabolites in root exudates of forbs. PCA was performed with the 138 forb samples for which semi-polar metabolite data were also available. Colours represent species (see legend).

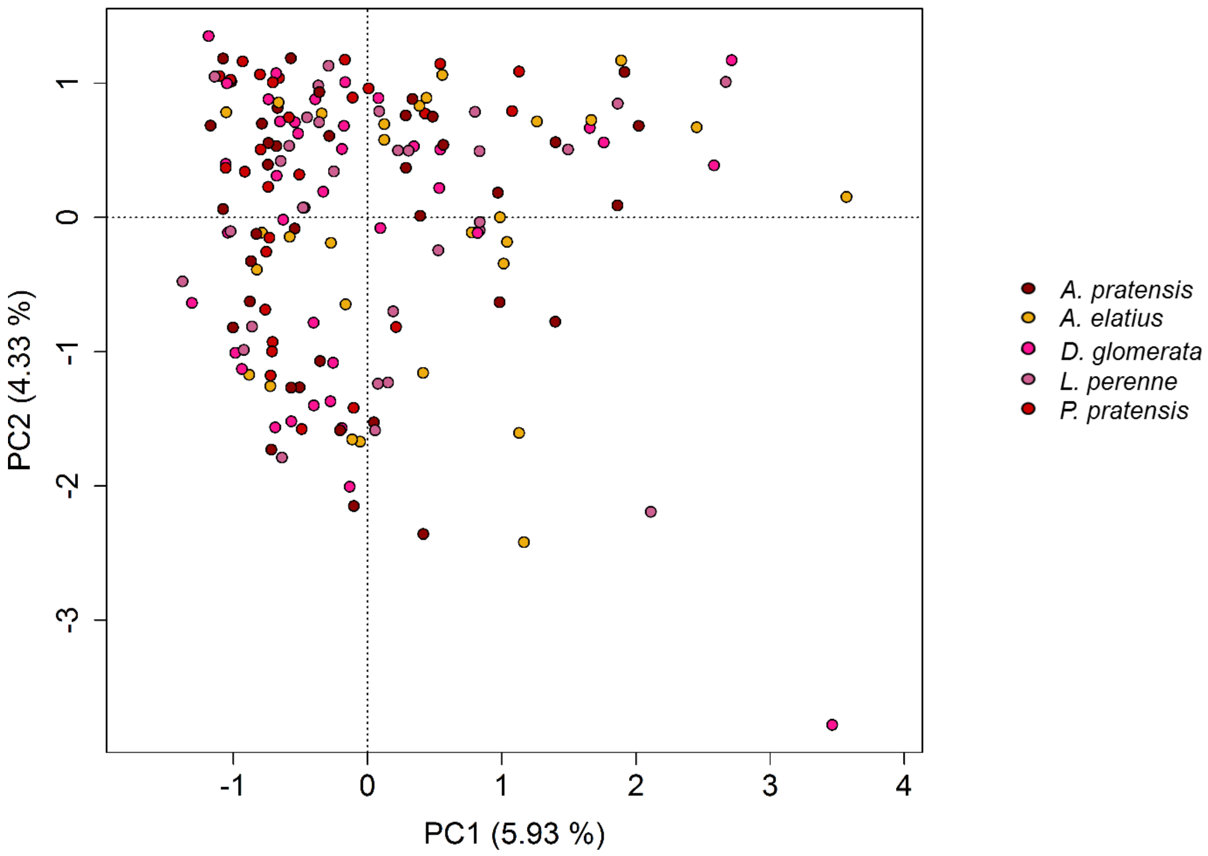

Figure A6: Principal component analysis of semi-polar metabolites in root exudates of grasses. PCA was performed with the 164 grass samples for which semi-polar metabolite data were also available. Colours represent species (see legend).

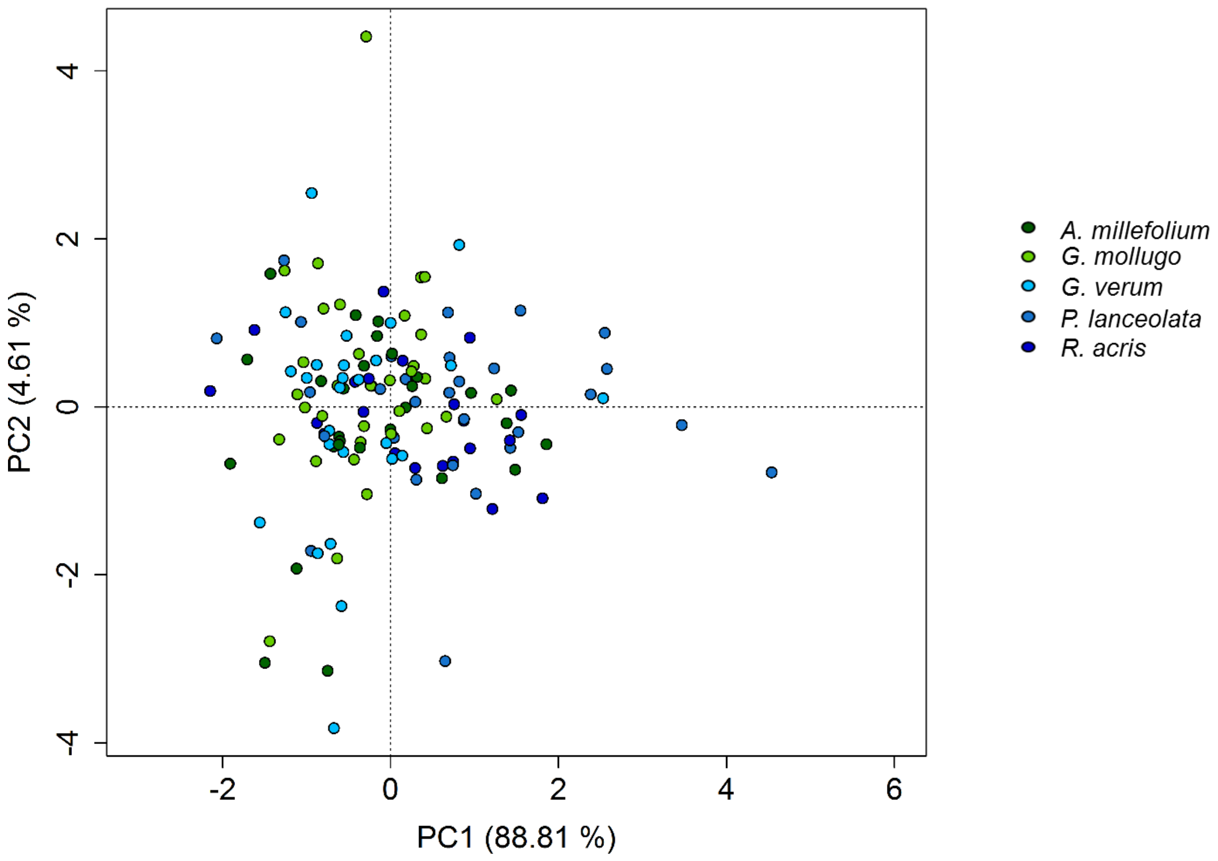

Figure A7: Principal component analysis of plant functional traits of forbs. PCA was performed with the 138 forb samples for which trait data were also available. Colours represent species (see legend).

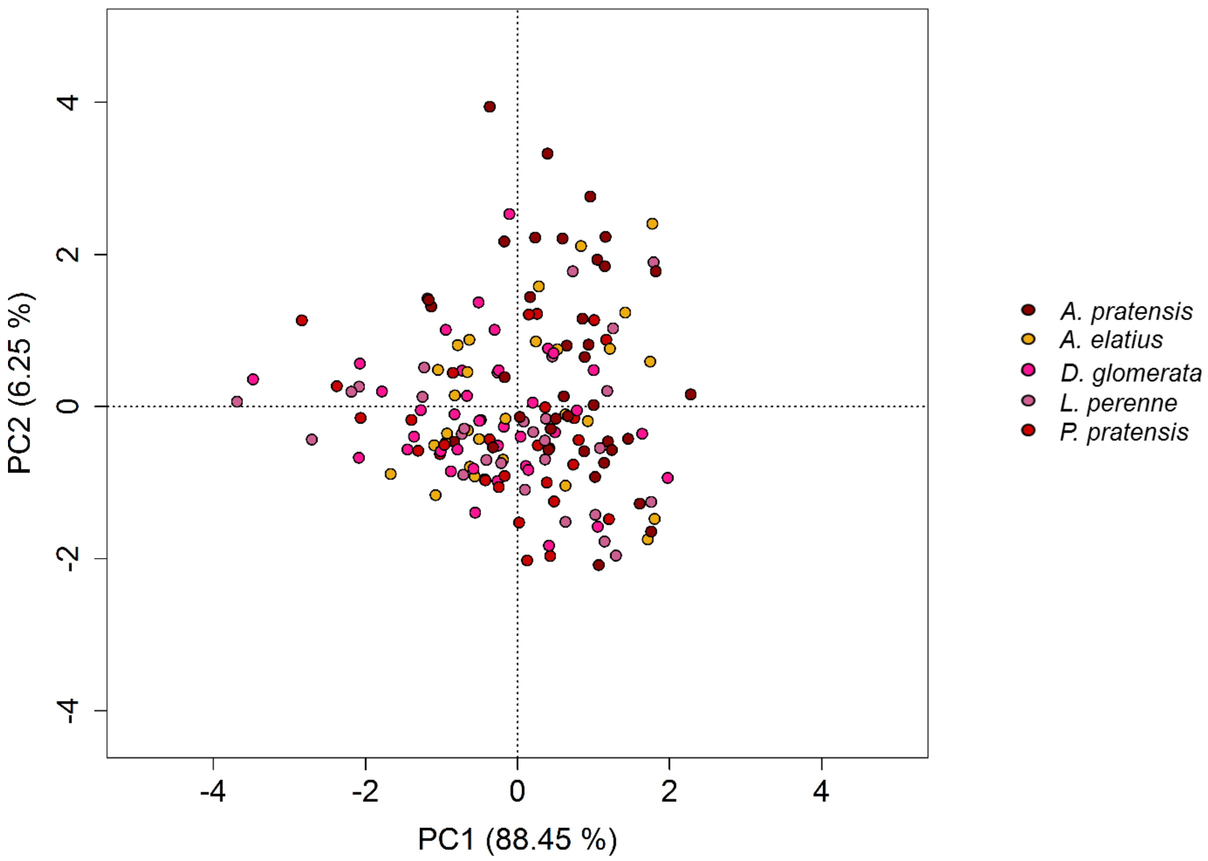

Figure A8: Principal component analysis of plant functional traits of grasses. PCA was performed with the 164 grass samples for which trait data were also available. Colours represent species (see legend).

Table A1: Identifier fragments and characteristic masses. The table contains all neutral losses and fragment ions which were used for classification of compounds in table A2.

**identifier fragments**	**masses (m/z)**
neutral losses	
Methoxylated aromatic compounds 30.0454	30.0454
Polyketides 42.0117	42.0117
Imin /Cholin ester 59.0133	59.0133
O-sulfated compounds 79.9615	79.9615
Hexose (-H_2O)162.0484	162.0484
Hexose 180.0581	180.0581
Fragment ions	
Carboxylic acid 89.0284	89.0284
Phosporous group ($H_2PO_4^-$) 96.9582	96.9582
Sulfate fragment (HSO_4^-) 96.9590	96.9590
Sugar fragment 101.023	101.023
Sugar fragment 113.0227	113.0227
Coumaroyl fragment 119.0499	119.0499
Caffeoyl fragment 135.0432	135.0432
Salicylate fragment 137.0231	137.0231
Caffeoyl fragment 145.0283	145.0283
Sinapoyl fragment 149.0226	149.0226
Dihydroxybenzoic acid, Gentisate 153.0087	153.0087
Phosphorylated fragment 158.9942	158.9942
Desoxyglycosylated fragment 161.045	161.045
Coumaroyl fragment 163.0359	163.0359
Caffeoyl fragment 163.0491	163.0491
Rhamnoside fragment 163.0715	163.0715
Sinapoyl fragment 164.0452	164.0452
Feruloyl fragment or Methoxy-coumarin fragment 175.038	175.038
Ferulic acid fragment 178.0264	178.0264
Caffeoyl fragment 179.0338	179.0338
Hexose fragment 179.0561	179.0561
Sinapic acid fragment 179.0713	179.0713
Sinapoyl-related fragment 190.0182	190.0182

Quinic acid fragment 191.057	191.057
Ferulic acid fragment 193.0514	193.0514
Sinapoyl-related fragment 205.0538	205.0538
Fraxetin fragment 207.0333	207.0333
Glycosides_1-O-methyl-β-D-glucuronate fragment 207.0657	207.0657
Sinapic acid fragment 223.0602	223.0602
Aglycon of sesquiterpene glycosides 239.0293	239.0293
Phosphatidylcholine headgroup 255.0144	255.0144
Kaempferol backbone fragment 255.029	255.029
Hexose phosphate 259.0262	259.0262
Naringenine fragment 271.0634	271.0634
Kaempferol backbone 283.0553	283.0553
Kaempferol, Luteolin, Cyanidin fragment 284.0352	284.0352
Kaempferol fragment, Anthocyanidine-backbone 285.0397	285.0397
Catechin/Epicathechin 289.0995	289.0995
Jasmonate fragment 291.1991	291.1991
Quercetin fragment 299.0585	299.0585
Quercetin fragment 300.0661	300.0661
Quercetin fragment 301.0291	301.0291
2xMe-Kaempferol fragment 313.0761	313.0761
Me-Quercetin fragment 315.0592	315.0592
Disaccharide fragment 323.0947	323.0947
2xMe-Kaempferol fragment 327.0813	327.0813
2xMe-Quercitin fragment 329.0709	329.0709
Caffeoyl-Glucose fragment 341.0821	341.0821
4xMe-Kaempferol fragment 341.1084	341.1084
Succrose fragment 341.1454	341.1454
2xMe-Myricitin fragment 345.0545	345.0545
Esculetin(4-O-8)G fragment 355.0729	355.0729
3xMe-Myricetin fragment 359.0822	359.0822
4xMe-Myricitin fragment 373.0995	373.0995
Esculetin(4-O-8)S fragment 385.0927	385.0927
Sesquiterpene diglycoside malonylated fragment 443.2687	443.2687
Agnuside fragment 465.1391	465.1391

Table A2: Putative classification of the significantly species specific semi-polar metabolites

The table contains no. of metabolites, species, retention time (RT), m/z value, p value for significance of occurrence in single species, type of adduct, putative elemental composition, putative classification, observed neutral losses and fragment ions upon CID, whereas identifier ions (see table S2) were marked in bold. * = precursor ion in addition to fragment ions and neutral losses used for annotation of compound. All measurements were obtained in negative ionisation mode.

No.	**Species**	**RT [min]**	**m/z**	**p-value**	**Type of adduct**	**Putative Elemental composition**	**Putative Class**	**Fragment ions and neutral losses detected in CID (identifier masses in bold)**	
								**Neutral losses**	**Fragment ion**
1	*A. millefolium*	2.62	409.04451	$4{,}55x10^{-12}$	[M-H]-	C12H16N3O11S	Unclassified, sulfated	198.0589, 212.0011, 312.0866, 227.0244	210.9914, 197.0441, **96.9582,** 182.0228
2	*A. millefolium*	3.11	619.18815	$6{,}03x10^{-03}$	[M-H]-	C26H36O17	Flavonoid, glycosylated	474.1619, 502.1567, 278.0821, 506.1664, 456.1454	145.0283, 117.0312, **341.1084**, **113.0227**, **163.0491, 119.0499, 179.0561**
3	*A. millefolium*	4	421.1582	$8{,}52x10^{-04}$	[M-H]-	C25H27O4P	Glycoside	184.105, **180.0581**, 308.1367, 200.0463, **162.0484**	237.0533, 241.1095, **113.0227**, 221.1108
4	*A. millefolium*	4.43	413.14458	$2{,}14x10^{-03}$	[M-H]-	C14H26N2O12	Unclassified	221.0659, 220.0538	192.0732, 193.0845

5	*A. millefolium*	4.44	559.27218	$7,13x10^{-04}$	[M-H]-	C27H44O12	Phenylpropa-noid, glycosylated	208.0522, 360.2372, 207.0535, 307.0735, 349.1792, 208.1591, 358.2673, 362.1566, 259.0752, 334.2148, 283.0589, 360.1399, 476.3299, 378.1646, 353.2364, 344.1622	351.2203, 199.0348, 252.1994, 210.0863, 351.1057, 201.0071, 197.1206, 300.1976, 225.0548, 276.2086, 199.1277, 82.9457, 206.0411, 181.1174, 215.1089, **113.0227, 179.0713, 175.038**
6	*A. millefolium*	4.64	491.21285	$3,90x10^{-04}$	[M-H]-	C22H36O12	Glycoside	192.0655, 378.1885, 330.1615, 244.1306	299.146, **113.0227**, **161.045**, 247.0785
7	*A. millefolium*	4.77	509.22072	$7,91x10^{-14}$	[M-H]-	C22H38O13	Glycoside	178.0490, 276.1558, 348.1760, 307.1988, 396.1989, 384.1977, 177.0437, 366.1900, 350.1904, 306.1959, 46.0083	331.1782, 233.0644, **161.0450**, 202.0248, **113.0227**, 125.0235, 143.0330, 159.0343, 203.0295, 463.2102, **101.0230**
8	*A. millefolium*	4.82	503.17797	$3,15x10^{-16}$	[M-H]-	C22H32O13	Glycoside	355.1230, 340.1032, 378.1529,	148.0485, **163.0715**, 125.0235,

								390.1506, 354.1208, 270.1108	**113.0227**, 149.0596, 233.0644
9	*A. millefolium*	4.86	517.18406	1,24x10^{-07}	[M+HCOOH-H]-, [M-H]-	C23H28N4O7	Unclassified	369.1411, 354.1208, 270.1108, 374.1600	148.0485, 163.0715, 247.0785, 143.0330, 125.0235, 113.0227
10	*A. millefolium*	4.86	537.16869	4,03x10^{+00}	[M-H]-	C32H26O8	Polyphenole, Hydroxy-cinnamicacid	390.1186, 199.0862, 214.1195, 344.1622, 410.1210, 423.1057, 279.0868, 243.0697, 350.0774, 428.1306, 342.0776, 344.1192, 241.5948, 349.0797, 399.1010	147.0460, 338.0599, 323.0545, 192.9962, 127.0393, 258.0749, 114.0531, 294.0901, 187.0943, 109.0263, 195.0830, 193.0407, 295.5674, 188.0813, **163.0359**,
11	*A. millefolium*	4.89	547.20258	1,29x10^{-27}	[M+HCOOH-H]-, [M-H]-, [M+Cl]-	C23H34O12	Phenylpropa-noid	354.1208, 369.1411, 422.1756, 384.1622, 353.1117, 300.1251	193.0845, 178.0677, 125.0235, **163.0359**, 194.0906, 247.0785, **179.0713**
12	*A. millefolium*	4.89	647.29147	6,91x10^{-03}	[M-H]-	C25H50N3O14S	Unclassified	502.2059	145.0942

13	*A. millefolium*	4.9	689.29637	$7,57x10^{-13}$	[M-H]-	$C_{30}H_{48}N_3O_{15}$	Unclassified	466.0796, 481.1047, 465.0809, 448.1388	223.0602, 208.0390. 224.0616, 241.0031
14	*A. millefolium*	4.91	307.10868	$2,40x10^{-05}$	[M-H]-	$C_{10}H_{18}N_3O_8$	Unclassified	15.8486, 103.0292, 88.1494, 37.6603, 46.6628, 163.5876, 225.2302, , 66.1459, 258.6425, 203.4779, 244.6099, 125.9688, 134.1051, 162.8712, 46.7496	307.0735, 291.2591, 204.0788, 218.9630, 143.5200, 260.4449, 269.4474, 81.8818, 48.4640, 240.9707, 62.4977, 103.6298, 172.9970, 181.1363, 144.2365
15	*A. millefolium*	5.02	487.18126	$3,00x10^{-21}$	[M-H]-, [M+Cl]-	$C_{22}H_{32}O_{12}$	Glycoside	326.1378, 362.1566	**113.0227**, 115.0035, **161.045**
16	*A. millefolium*	5.13	401.13835	$1,96x10^{-09}$	[M-H]-	$C_{18}H_{26}O_{10}$	Phenylpropa-noid	208.0811, 223.0694, 224.1493, 307.1988, 162.1258, 154.0709, 207.0731, 202.0608, 128.9881, 61.9278, 289.3524, 238.0954, 160.0478, 214.1071, 327.7048	193.0656, 178.0677, 176.9997, 93.9404, 247.0785, 239.0293, 194.0562, 199.0748, 272.1528, 339.1974, 111.7927, **163.0491**, […]-, **373.0995**, 267.0266,

17	*A. millefolium*	5.13	469.1706	$4{,}15x10^{-14}$	[M-H]-, [M+HCOOH-H]-	C22H30O11	Unclassified	252.044, 208.0598, 251.044	217.1226.261.1088, 218.1309
18	*A. millefolium*	5.19	471.18573	$5{,}74x10^{-07}$	[M-H]-	C23H36O6S2	Unclassified	254.0618, 208.0598, 252.0539	217.1226.263, 1309, 219.1300, 66.2180, 1309, 261.1088
19	*A. millefolium*	5.36	452.19105	$6{,}58x10^{-05}$	[M-H]-	C23H27N5O5	Unclassified	254.0618, 208.0598, 252.0539, 210.0718	217.1226, 263.1309, 219.1366, 218.1309, 261.1088
20	*A. millefolium*	5.51	499.17737	$3{,}91x10^{-09}$	[M-H]-	C23H32O12	Phenylpropa-noid	321.1186	178.0677, 193.0845, **163.0359, 179.0713**, 194.0906
21	*A. millefolium*	5.51	567.18974	$3{,}90x10^{-04}$	[M-H]-	C33H28O9	Glycoside	**324.1046**	243.0664, 199.0748
22	*A. millefolium*	5.54	477.22204	$2{,}19x10^{+00}$	[M-H]-	C21H34O12	Phenylpropa-noid	240.1343, 253.1528, , 236.1168, 224.1493, 209.1373, 105.1423, 284.1416, 278.1279, 135.5813, 212.0921,	237.0799, 224.0455, 267.0680, 241.0880, 253.0495, 268.0693, 372.0577, 193.0656, 199.0748, 341.6239,

								176.0448, 308.1529, 221.1056, 208.0347, 223.2450	265.1088, 301.1637, 169.0459, […]-, **137.0331, 153.0087**
23	*A. millefolium*	5.95	445.23445	1,10x10^{-13}	[M-H]-	C29H34O4	Glycoside	160.0283, 253.1925, 238.1699, 295.2107, 244.1189, 332.2063, 207.0535, 343.1352, 204.0904, 334.1868	285.1942, 192.0411, **207.0657**, 150.0288, 201.1082, **113.0227**, 238.1843, 102.1033, 241.1420, 111.0498
24	*A. millefolium*	6.1	508.24348	2,37x10^{-18}	[M-H-H]-2	C50H74N4O18	Unclassified	267.1878, 208.1432, 266.1808	241.0499, 300.0969, 242.0526
25	*A. millefolium*	6.15	451.15528	4,87x10^{+00}	[M-H]-	C22H28O10	Glycoside	326.1378, 338.1439, 328.1141, **324.1046**, 350.1334	125.0235, **113.0227**, 123.0437, 127.0599, **101.0230**
26	*A. millefolium*	6.5	289.12929	6,91x10^{-03}	[M-H]-	C13H22O7	Unclassified		
27	*A. millefolium*	7.22	303.1444	7,23x10^{-02}	[M-H]-	C15H20N4O3	Unclassified	174.0497, 190.0396, 146.0531	129.0969, 113.1028, 157.0894

28	*A. millefolium*	8.94	286.18069	$9{,}36\times10^{-19}$	[M-H]-, [M+HCOOH-H]-	C18H25NO2	Unclassified	120.0623	286.1750, 166.1286
29	*A. pratensis*	3.3	347.04423	$1{,}05\times10^{-03}$	[M-H]-	C27H8O	Polyphenole, Hydroxy-cinnamic acid	153.9980, 213.0175, 169.0206, 152.9913, 197.9887	**193.0514**, 134.0357, **178.0264**, 194.0562, 149.0596
30	*A. elatius*	3.69	417.1763	$3{,}61\times10^{-04}$	[M-H]-	C26H26O5	Glycoside	206.0316, **180.0581**, 224.0503, 218.1546, 254.0618, 289.1342, 287.0805, 223.0400, 302.1690, 232.0961, 206.1083, 188.9991, 250.1350, 204.0904, 150.0739	211.1394, 237.1120, 193.1251, 199.0149, 163.1143, 128.0329, 130.0882, 194.1225, 115.0035, 185.0768, 211.0557, 228.1714, 167.0372, 213.0870, 267.0962
31	*A. elatius*	4.92	571.22054	$3{,}10\times10^{+00}$	[M-H]-	C27H41O11P	Unclassified, phosphoryla-ted, glycosylated	336.1860, 458.2057, 201.1309, 306.0875, 326.0443, 347.1402, 378.1885, 332.1020,	235.0498, **113.0227**, 370.0970, 265.1502, 245.1879, 224.0941, 193.0407, 239.1345,

								323.1052,<br>346.1186,<br>384.1333,<br>322.0820,<br>338.1194,<br>432.2342,<br>446.1397	248.1310,<br>225.1095 ,<br>187.0943,<br>249.1596,<br>233.1143,<br>139.0060,<br>125.0969,<br>311.1003,<br>**179.0561**,
32	*A. elatius*	5.37	545.25984	$1,04x10^{-01}$	[M-H-H]-2	C59H80O19	Phenylpropa-noid, glycosylated	288.1951,<br>435.2376,<br>351.2195,<br>333.0900,<br>331.1918,<br>397.2122,<br>444.1984,<br>299.1887,<br>400.3703,<br>271.0627,<br>282.1632,<br>346.2394,<br>420.1722,<br>434.1783,<br>454.3455,<br>267.7391,<br>404.1959,<br>251.1830<br>234.2122,<br>355.2432,<br>201.1721	257.0639,<br>194.0360,<br>110.0155,<br>212.1623,<br>214.0614,<br>101.0548,<br>148.0485,<br>246.0590,<br>263.0813,<br>274.1905,<br>199.0149,<br>144.8829, […]-,<br>**190.0182**,<br>311.0494,<br>344.0910,<br>218.0779,<br>250.0742,<br>216.9399, […]-,<br>**373.0995**,<br>298.1836, […]-,<br>**163.0715**
33	*A. elatius*	5.62	577.25563	$8,53x10^{-02}$	[M-H-H]-2	C59H82NO22	Terpene	41.9252, 214.0391,<br>336.2472,<br>375.1691,<br>356.0985,<br>332.0615,	535.3287,<br>363.2249,<br>241.0031,<br>202.0803,<br>221.1555,

								372.1222, 331.1691, 344.1192, 298.1251, 426.1724, 310.1815, 338.0836	245.1879, 205.1253, 246.0945, 233.1330, 279.1302, 151.0798, 267.0680, **239.0293**
34	*A. elatius*	5.62	600.26278	1,04x10^{-02}	[M-H]-	C24H59NOS7	Unclassified, glycosylated, sulfated	379.1935, 62.9272, 499.2358, 184.5433, 184.0594, 439.2198, 34.9453, 317.1984, 351.2195, 441.2322, 246.1422, 326.1542, 305.1261, 371.1090,	221.0671, 537.3438, **101.0230**, 415.7166, 416.1974, 161.0450, 565.3227, 283.0553, 249.0378, 159.0343, [….]-, **113.0227**, [….]-, 554.3342, **96.9701**,
35	*A. elatius*	5.69	567.26537	1,04x10^{-02}	[M-H]-	C25H44O14	Unclassified, glycosylated	176.0448, 338.0836, 328.1309, 268.0672, 238.0502, 270.0939, 45.9238, 314.0875, 250.0623, 216.0324, 454.2374, 352.1821, 232.1181, 194.0543, 237.061	391.2188, 229.1727, 239.1345, 299.2021, 329.2184, 297.1781, 521.3447, 253.1796, 317.2055, 351.2203, **113.0227**, 215.0864, 373.2153,

									335.1512, 330.2065
36	*A. elatius*	5.92	563.31879	1,03x10^{+00}	[M-H]-	C37H44N2O3	Terpene, glycosylated	176.0448, 338.0836, 328.1309, 268.0672, 238.0502, 270.0939, 45.9238, 314.0875, 250.0623, 216.0324, 454.2374, 352.1821, 194.0543, 232.1181, 237.0610, 452.2092, 249.9808, 346.1655,	391.2188, 229.1727, 239.1345, 299.2021, 329.2184, 297.1781, 521.3447, 253.1796, 317.2055, 351.2203, **113.0227**, 215.0864, 373.2153, 335.1512, 330.2065, 115.0550, 221.1108, **443.2687**
37	*A. elatius*	6.76	975.50862	1,06x10^{-06}	[M+HCOOH-H]-, [M-H]-	C47H78O18	Diglycoside	754.4519, 208.0598, 712.4419, 207.0535, 502.1567, 370.1182, 814.4722, 796.4613, 652.4214,	221.0671, 767.4565, 263.0813, 473.3597, 605.4013, 161.0450, 179.0561, **323.0947**, 143.0330,

								832.4821, 862.4923, 753.4483,	**113.0227**, 222.0683,
38	*A. elatius*	7.57	547.32609	1,06x10^{-05}	[M-H]-	C31H48O8	Unclassified, Aromatic acid	43.9839, 42.9853, 234.1019, 260.1177, 338.2024, 296.1606,	503.3342, 313.2108, 287.1985, 209.1166, 251.1720,
39	*A. elatius*	8.09	299.07602	7,56x10^{-01}	[M-H]-	C16H12O6	Flavonoid	89.9010, 92.0235, 108.0220, 59.0133, 107.0533, 15.0215, 27.9912, 123.0423,	209.1502, **207.0333**, 191.0361, 240.0408, 192.0070, **284.0352**, **271.0634**, 176.0118,
40	*D. glomerata*	3.93	245.13851	3,13x10^{-04}	[M-H]-	C10H20N3O4	Unclassified	122.0567, 104.0515, 130.9931, 134.0537, 22.2797, 62.0349, 90.0298, 178.4211, 74.0352, 115.0552, 95.1331, 110.1478, 59.1660, 86.4543, 86.8307	123.0811, 141.0882, 114.1437, 111.0800, 222.8570, 155.1107 , 183.1058, 66.7238, 130.0882, 171.1040, 150.0037, 134.9882, 158.6784, 185.9703, 158.3060
41	*G. mollugo*	3.92	611.24752	3,18x10^{-06}	[M-H]-	C27H40N4O12	Unclassified	360.1238, 404.1125,	251.1261, 207.1380,

								359.1208, 403.1087,	252.1254, 208.1377
42	*G. mollugo*	4.08	509.21506	2,26x10^{-08}	[M-H]-	C22H38O13	Glycoside	178.0490, 348.1760, 177.0437, 396.1989, 276.1558, 46.0083	331.1782, **161.0450**, **113.0227**, 233.0644, 463.2102
43	*G. mollugo*	4.13	377.17599	1,71x10^{-07}	[M-H]-	C17H30O9	Flavonoid, glycosylated	174.1894, 189.212, 243.1335, 163.0362, 78.1171, **162.0484**, 191.1474, 79.0928, 170.0986, 160.1037, 204.0904, 75.1080, 33.4871, 132.1216, 168.2815, 204.9187,	202.9945, 187.9662, 134.0470, 214.1438, **299.0585**, 215.1294, 186.0360, 298.0767, […]-, 188.0625, 217.1526, 222.0426, 242.0731, 253.1076, 313.0420, **113.0227**
44	*G. mollugo*	4.78	467.11897	4,09x10^{-08}	[M-H]-	C21H24O12	Unclassified	265.0892, 264.0863	202.0248, 203.0295
45	*G. mollugo*	5.73	419.13406	5,27x10^{-15}	[M-H]-	C21H24O9	Flavonoid	207.0535, 163.0602, 206.0509, 192.1030, 162.0590	212.0847, 256.0754, 213.0870, 227.0351, 257.0800, **255.0290**
46	*G. mollugo*	5.75	563.1378	8,96x10^{-01}	[M-H]-	C26H28O14	Unclassified	295.1032, 294.0928, 310.0864,	268.0361, 269.0481, 253.0495,

								312.1032, 340.0923, 323.0883	251.0338, 223.0434, 240.0408
47	*G. mollugo*	6.09	297.04047	9,71x10^{-21}	[M-H]-	C14H8N3O5	Unclassified	43.9916, 87.0083, 42.9853	253.0495, 210.0334, 254.0539
48	*G. mollugo*	6.1	253.05029	2,27x10^{-14}	[M+K-H]-, [M+Na-H]-	C10H15O5	Polyketide, Aromatic acetate	43.0179, **42.0117**	210.0334, 211.0377
49	*G. mollugo*	6.14	283.05943 *	8,68x10^{-07}	[M-H]-	C16H12O5	Flavonoid	30.0051, 44.0247, 73.0253, 72.0167, 29.0036, 43.0179, 15.0215	253.0495, 239.0293, 210.0334, 211.0377, 254.0539, 240.0408, 268.0361, **283.0553**
50	*G. mollugo*	6.4	691.23246	2,15x10^{-01}	[M-H]-	C33H40O16	Glycoside	**324.1046**, 323.1052, 339.1370, 382.1837	367.1275, 352.0933, 309.0403
51	*G. mollugo*	6.83	489.17542	3,98x10^{-13}	[M-H]-, [M+Cl]-	C26H26N4O6	Unclassified	205.0779, 264.0863, 260.1284, 282.0966, 248.1243, 204.0625, 220.0992	284.1042, 225.0840, 229.0428, 207.0831, 241.0499, 285.1061, 269.0796
52	*G. mollugo*	7.74	269.0503	3,10x10^{-06}	[M-H]-	C16H6N4O	Unclassified		

53	*G. verum*	6.24	299.02009	$4,41x10^{-01}$	[M-H]-	C15H8O7	Flavonoid	43.9839, 87.9814, 44.9635, 88.9932, 71.9868, 18.0110, 42.9853	**255.0290**, 211.0377, 254.0539, 210.0334, 227.0351, 280.9980, 256.0316
54	*G. verum*	7.62	311.00608	$2,11x10^{-01}$	[M-H]-	C17H12O6	Polyketide, aromatic acetate	43.0179, **42.0117**	268.0361, 269.0481
55	*Galium spp.*	2.68	241.07151	$9,28x10^{-07}$		C14H12NO3	Unclassified, Imin fragment	62.0006, **59.0133**	
56	*Galium spp.*	2.68	449.12732	$6,71x10^{-38}$	[M+HCOOH-H]-, [M-H]-, [M+Cl]-	C17H24O11	Phenylpropa-noid, glycosylated	310.0864, 208.0522, 240.0812, 206.0395, 348.1023, 258.0909, 322.0820, 254.0618, **324.1046**, 226.0637, 238.0668, 328.0940, 288.1027, 338.1194	139.0371, 241.0726, 209.0445, 243.0891, **101.0230**, 191.0361, 127.0393, 195.0663, 125.0235, **223.0602**, 211.0694, 121.0294, **205.0538, 119.0499**
57	*Galium spp.*	2.96	355.09956 *	$5,88x10^{-27}$	[M-H]-	C16H20O9	Polyphenole, Hydroxycinnamic acid	**162.0484**, 221.0560 , 206.0316, 177.0710, 161.0488	193.0514, 134.0357, 149.0596, 178.0264, 194.0562, **113.0227,**

									**255.0290, 179.0561**
58	*Galium spp.*	3.05	391.15882	3,56x10^{-08}	[M-H-H2O]-, [M+Na-H]-	C17H30O11	Flavonoid, glycosylated	198.1012, 190.1682, 168.1210, 272.1215, 280.0749, 76.1030, 131.0847, 160.0283, 62.0765, 75.0302, 343.5816, 189.0825, 278.1279, 174.0331, 144.1086	**193.0514**, 200.9925, 223.0434, 119.0333, 111.0800, 315.0592, 260.0752, 231.1375, **329.0709**, 316.1216, 47.5766, 202.0803, 217.1226, **178.0264, 355.0729**
59	*Galium spp.*	3.08	389.11408	7,53x10^{-11}	[M-H]-	C16H22O11	Polyphenole, Hydroxycinn amicacid	188.1065, 268.0785, 268.0449, 224.0395, 266.0629, 250.1043, 174.0813, 247.1258, 130.9602, 246.0678, 196.0587, 174.0071, 234.0257, 181.0460, 127.9720,	200.9925, 121.0294, 121.0645, 165.0557, 123.0437, 139.0060, 386.1923, 215.0292, 141.9803, 258.1450, 143.0451, **193.0514**, 215.1089, 155.0812, 208.0636, 261.1441

60	*Galium spp.*	3.11	583.17685	1,47x10^{-14}	[M-H]-	C19H36O20	Flavonoid, glycosylated	410.1052,<br>451.1437,<br>248.0447,<br>291.0576,<br>371.0893,<br>338.0907,<br>468.1725,<br>456.1361,<br>247.0408,<br>453.1068,<br>286.0631,<br>392.1121,<br>300.1251,<br>382.0773,<br>409.0971,<br>352.1153,	173.0767,<br>132.0300,<br>335.1225,<br>292.1146,<br>212.0847,<br>245.0744,<br>115.0035,<br>127.0393,<br>336.1404,<br>**283.0553**,<br>**113.0227**,<br>**345.0545,**<br>**161.0450**,<br>**299.0585,**<br>**313.0761,**<br>**163.0359,**	[…]-,<br>[…]-,<br>[…]-,
61	*Galium spp.*	3.26	671.20884	5,26x10^{-11}	[M-H]-	C25H40N2O19	Unclassified	371.1181,<br>370.1106,<br>430.1709,	300.0969,<br>301.1032,<br>241.0499,	
62	*Galium spp.*	3.48	539.23314	3,56x10^{-08}	[M+HCOOH-H]-, [M-H]-	C22H38O12	Coumarin, glycosylated	208.0522,<br>378.1885,<br>426.2025,<br>438.2069,<br>294.1636,<br>332.2063,<br>428.2173,<br>276.1458,<br>396.1989,<br>400.2250,	331.1782,<br>**161.0450**,<br>**113.0227**,<br>**101.0230**,<br>245.0744,<br>**207.0333**,<br>**111.0096**,<br>263.0813,<br>143.0330,<br>139.0060,<br>175.0228	
63	*Galium spp.*	3.49	433.13439	5,80x10^{-07}	[M-H]-	C18H26O12	Glycoside	226.0637,<br>310.0864,<br>332.1020,	**207.0657**,<br>123.0437,<br>**101.0230,**	

								208.0522, 225.0871,	225.0840, 208.0390,
64	*Galium spp.*	3.69	377.1741	9,37x10^{-17}	[M-H]-	C20H29NO4P	Flavonoid, glycosylated	162.0831, 205.1117, 171.1213, 157.0813, 151.1725, 238.5506, 48.1162, 194.0887, 237.2491, 303.0122, 317.1016, 78.1523, 118.1453, 134.1254	215.0864, 172.0551, 206.0663, 220.0881, 138.6255, 226.0036, **329.0709**, **285.0397**, 183.0870, 139.9273, 74.1584, **259.0262**, 60.0759, […]-, **271.0634**
65	*Galium spp.*	3.7	345.15503	2,42x10^{-05}	[M-H]-	C16H26O8/C17H22N4O4	Phenylpropa-noid/ Agmatine	215.0950, 193.0944, 180.0441, 117.0119, 276.5944, 212.7553, 65.9882, 88.0636, 148.1353, 149.1106, 118.1453, 168.0674, 229.0701, 196.0587, 88.9490	130.0592, 152.0418, 165.0905, 228.1451, 68.5488, 132.3910, 196.0337, 257.0800, 197.0184, 279.1562, 177.0788, 226.9998, 116.0722, **193.0514, 241.0031**
66	*Galium spp.*	3.77	553.24547	1,57x10^{-20}	[M-H]-	C31H38O9	Flavonoid	406.1987, 222.0578, 404.2148, 358.1354, 371.1988, 347.1727,	147.046, 331.1782, **149.0226**, 195.1101, 206.0663, 182.0439, 146.3428,

								406.9088, 339.1885, 296.2380, 431.2060, 60.3026, 354.1740, 334.1688,	257.0001, 214.0614, 122.0410, […]-, […]-, **313.0761,**
67	*Galium spp.*	3.78	499.19377	2,17x10^{-27}	[M-H]-	C34H28O4	Flavonoid	296.1893, 214.1071, 392.1121, 286.1062, 259.1703, 276.0535, 277.0726, 314.0668, 272.0884, 248.1432, 218.1926, 418.0122, 293.1743	202.9945, 285.0761, 107.0821, 213.0870, 223.1343, 240.0148, 185.1181, 222.1187, 227.0937, 251.0494, 280.9980, 224.0616, […]-, **359.0822**
68	*Galium spp.*	4.13	493.17993	1,44x10^{-13}	[M-H]-	C22H30N4O9	Unclassified	226.1253, 291.1656,	267.068, 202.0248,
69	*Galium spp.*	4.18	509.22311	3,64x10^{-11}	[M-H]-	C29H34O8	Glycoside	178.0490, 348.1760, 177.0437, 396.1989, 276.1558, 46.0083,	331.1782, **161.0450**, **113.0227**, 233.0644, 463.2102, **101.0230**
70	*Galium spp.*	4.25	899.3127	3,56x10^{-08}	[M-H]-	C36H56N2O24	Unclassified	402.19, 697.2910, 686.2632, 401.1878, 696.2861,	497.1286, 202.0248, 213.0493, 203.0295,

								672.2834, 498.1376,	227.0351, 401.1807,
71	*Galium spp.*	4.36	389.12107	$1,36x10^{-01}$	[M-H]-	C24H24N4O5	Unclassified	238.0502, 178.0234, 240.0202, 222.0145, 196.0382, 209.1373,	209.1166, 269.1404, 207.1380, 225.155, 251.1261, 238.0251,
72	*Galium spp.*	4.4	447.16464	$3,96x10^{-13}$	[M+HCOOH-H]-, [M-H]-, [M+Cl]-	C32H36NO11	Unclassified	371.1181, 370.1182, 277.1296,	284.1042, 285.1061, 378.0986,
73	*Galium spp.*	4.67	549.25373	$4,88x10^{-14}$	[M-H]-	C25H42O13	Phenylpropa-noid, glycosylated	340.0923, 178.0364, 388.2034, 436.2201, 177.0437, 448.2231, 316.1849, 406.2136, 339.0904,	209.1502, 371.2017, **161.045**, **113.0227**, 372.2127, **101.023**, 233.0644, 143.033, **191.0570**
74	*Galium spp.*	4.78	437.10837	$1,37x10^{-09}$	[M-H]-	C20H22O11	Phenylpropa-noid	258.0723, 242.0462, 164.9614, 206.0316, 185.0637, 220.1309, 198.0589, 235.0903, 168.1210, 228.0864, 255.9914	**179.0338**, 195.0663, 272.1528, 231.0859, 216.9834, 252.0411, 239.0477, 202.0248, 209.0290, 124.0170,

									181.1174, 268.9853
75	*Galium spp.*	4.94	563.13922	$2,38x10^{-26}$	[M-H]-	C27H24N4O10	Unclassified	312.1032, 294.0928, 311.0984, 326.0844, 298.0878, 293.0882,	251.0338, 269.0481, 252.0411, 237.0533, 265.0429, 270.0456,
76	*Galium spp.*	5.07	625.17515	$1,60x10^{-16}$	[M-H]-	C26H32N3O15	Unclassified	369.1042, 413.0866, 412.0838, 368.0952,	256.0754, 212.0847, 213.0870, 257.08,
77	*Galium spp.*	5.15	609.1758	$1,37x10^{-09}$	[M-H]-	C28H34O15	Phenylpropa-noid, glycosylated	488.1460, 340.1277, 478.1328, 460.1191, 342.1113, 358.1453, 496.1614, 418.1268, 487.1496, 495.1459, 402.1900	121.0294, 269.0481, 131.0515, 149.0596, 267.0680, 251.0338, **113.0227**, **191.0570**, 122.0301, […]-, **175.0380, 161.0450**
78	*Galium spp.*	5.28	595.16638	$5,26x10^{-11}$	[M-H]-	C25H30N3O14	Unclassified		
79	*Galium spp.*	5.38	275.0922	$2,54x10^{-45}$	[M-H]-	C15H16O5	Unclassified	88.0496, 87.0449, 116.0442,	187.0375, 188.0456, 159.0343,
80	*Galium spp.*	5.47	595.16624	$1,49x10^{-39}$	[M-H]-	C27H32O15	Phenylpropa-noid, glycosylated	339.0904, 383.0827, 338.0907, 368.1377,	256.0754, 212.0847, 257.0800,

									227.0351, **191.0570**
81	*Galium spp.*	5.48	547.14532	1,57x10^{-20}	[M-H]-	C24H26N3O12	Unclassified	295.1032, 294.0928, 310.0864, 356.0899, 416.1069, 434.1299, 293.0978, 398.0991,	252.0411, 253.0495, 237.0533, 191.0570, 131.0346, 113.0227, 254.0539, 149.0439,
82	*Galium spp.*	5.73	463.12566	8,50x10^{-34}	[M-H]-	C23H20N4O7	Unclassified	207.0535, 251.0440, 250.0345, 206.0509, 236.0889,	256.0754, 212.0847, 213.0870, 257.0800, 227.0351,
83	*Galium spp.*	5.84	547.14493	6,52x10^{-23}	[M-H]-	C26H28O13	Unclassified	294.0928, 293.0882,	253.0495, 254.0539,
84	*Galium spp.*	5.85	431.13376	8,61x10^{-19}	[M-H]-	C20H22N3O8	Unclassified	207.0464, 163.0602, 218.1151, 222.0671, 206.0509,	224.0941, 268.0693, 213.0201, 209.0729, 225.0840,
85	*Galium spp.*	6.08	463.15973	1,93x10^{-41}	[M-H]-	C21H26N3O9	Unclassified	222.1078, 163.0602, 221.1056,	241.0499, 300.0969, 242.0526,
86	*Galium spp.*	6.18	507.23321	7,51x10^{-36}	[M-H-H]-2	C35H71N17O14PS	Glycoside	192.0877, 266.1808, 394.2140, 207.1353, 296.1606, 191.0838, 382.2117,	315.1512, 241.0499, **113.0227**, 300.0969, 211.0694, 125.0235,

								392.2306, 265.1807,	115.0035, 242.0526,
87	*Galium spp.*	6.25	239.0351	1,87x10^{-24}	[M-H]-	C15H4N4	Unclassified	27.9912, 43.9916,	239.0293, 211.0377, 195.0456,
88	*Galium spp.*	6.28	283.02542	2,29x10^{-05}	[M-H]-, [M-H-H2O]-	C16H4N4O2	Unclassified	43.9916, 71.9868, 42.9853, 87.9814,	239.0293, 211.0377, 240.0408, 195.0456,
89	*Galium spp.*	6.62	267.02998	1,87x10^{-24}	[M-H]-	C16H4N4O	Unclassified	43.9916, 71.9868, 42.9853,	223.0434, 195.0456, 224.0455,
90	*Galium spp.*	6.88	807.41276	1,37x10^{-09}	[M-H]-	C42H64O15	Flavonoid, glycosylated	478.1938, 482.2877, 694.3974, 678.3998, 602.3362, 650.4003, 522.3185, 409.3394, 489.3883, 503.1910, 652.3350, 591.3814, 520.3743, 477.1850, 552.3432, 479.3235, 448.7728, 624.3880, 668.4130, 393.2433	329.2184, 325.1251, **113.0227**, 129.0161, 205.0864, […]-, 183.0286, 139.0060, **315.0592**, 537.3438, 414.1778, […]-, **175.0228**, 261.0195, 175.0808, 60.1830, 475.3423, […]-, 251.1033, 123.4200, 116.0062, 77.3653, **289.0396**

91	*Galium spp.*	6.94	269.04756	$1,34x10^{-23}$	[M-H]-, [M-H-H2O]-	C15H10O5	Unclassified	18.011, 17.0095,	251.0338, 252.0411,
92	*Galium spp.*	7.03	253.05071	$8,61x10^{-19}$	[M-H]-	C13H8N3O3	Polyketide	15.0215, 43.0179, 14.0187, **42.0117**	238.0251, 210.0334, 239.0293, 211.0377,
93	*Galium spp.*	7.19	491.1554	$2,38x10^{-22}$	[M+HCOOH-H]-, [M-H]-, [M+Cl]-	C18H26N2O11	Unclassified	208.0598, 267.0734, 268.0785, 209.0635, 240.0812, 266.0629, 282.0966, 284.0717, 207.0535,	283.1019, 224.0941, 223.0748, 282.0856, 251.0756, 225.0840, 209.0729, 207.0831, 284.1042,
94	*Galium spp.*	7.22	313.0712	$8,36x10^{-02}$	[M-H]-	C17H14O6	Flavonoid	**30.0454**, 15.0215, 58.0398, 29.0422,	283.0176, 298.0496, 255.0290, **284.0352**, 299.0585
95	*Galium spp.*	7.66	**299.05504** *	$1,64x10^{-02}$	[M-H]-	C16H12O6	Flavonoid	87.9814, **30.0454**, 15.0215, 43.9916, 58.0398, 86.9764,	211.0694, 269.0070, **284.0352**, 255.0564, 241.0031, 212.0847, **285.0397, 299.0585**
96	*Galium spp.*	7.87	269.04591	$1,14x10^{-16}$	[M-H]-	C13H8N3O4	Unclassified	43.9916, 15.0215, 27.9912, 43.0179, 46.0083, 44.9985,	225.0548, 254.0189, 241.0499, 226.0282,

								15.999, 61.9693, 18.011, 59.0133,	223.0434, 224.0455, 253.0495, 207.0831, 268.0693, 251.0338, 210.0334,
97	*Galium spp.*	8.05	255.03005	1,03x10^{-11}	[M-H]-	C14H8O5	Flavonoid	**27.9912**	**255.0290**, 227.0351,
98	*Galium spp.*	8.67	328.05884	3,56x10^{-08}	[M-H]-	C20H11NO4	Unclassified, Imin fragment	102.0324, **59.0133**, 88.0155, 118.0246, 101.0244, 117.0119, 62.0349, 132.9201, 87.0083, 44.8614, 106.0308, 90.0298, 73.0361, 126.9422, 134.0225, 46.8822, 58.0105,	226.0282, 269.0481, 240.0408, 239.0293, 210.0334, 327.2024, 227.0351, 211.0377, 266.0199, 195.1414, 241.0499, 283.1922, 222.0222, 238.0251, **255.0290**
99	*Galium spp.*	8.79	301.10803	1,90x10^{-40}	[M-H]-	C15H16N3O4	Unclassified	101.0987, 113.0983, 69.0713, 100.0955, 141.0937,	200.0177, 188.0203, 232.0334, 201.0071, 160.0198,
100	*Galium spp.*	8.8	232.03736	2,49x10^{-22}	[M-H]-	C12H9O5	Unclassified	29.0036, 124.0171, 89.1289, 88.0496, 90.242, 111.4848, 134.0815,	203.0295, 108.0200, 142.9091, 231.1463,

								131.6694, 153.5161, 69.4696, 174.1599, 149.4254, 40.7212, 79.4318, 174.6441,	231.0269, 143.9877, 141.7874, 120.5614, 97.9631, 100.3686, 78.5295, 162.5684, 57.8822, 82.6151, 191.3154, 152.6063, 57.3881,
101	*Galium spp.*	9.87	367.11863	4,74x10^{-06}	[M-H]-	C21H20O6	Unclassified	58.0815, 70.0786, 57.0765, 15.0215, 69.0713,	309.0403, 297.0396, 310.0412, 352.0933, 298.0496,
102	*Galium spp.*	9.93	388.11854	1,64x10^{-02}	[M-H]-	C23H19NO5	Unclassified	180.0822, 179.072,	208.039, 209.0445,
103	*Galium spp.*	10.46	354.13392	1,64x10^{-02}	[M-H]-	C20H21NO5	Unclassified	146.0927, 145.0897,	208.039, 209.0445,
104	*L. perenne*	5.6	305.13879	5,58x10-^{03}	[M-H]-	C17H22O5	Phenylpropa-noid	96.0258, 198.1012, 144.0835, 100.0955, 102.0324, 62.0121, 103.0685, 88.0636, 103.0846, 197.0835, 102.0746, 80.0064, 76.0539	209.1166, 107.0503, 305.1371, 161.0598, **205.0538**, 203.1052, 243.1404, 202.0803, 217.0795, 202.0568, 108.0518, **163.0359**, **149.0226**
105	*L. perenne*	8.28	289.14387	4,61x10^{-02}	[M-H]-	C17H22O4	Phenylpropa-noid	62.0006, 43.9916, 60.9952, 170.0986, 60.0239, 182.0952,	227.144, 245.1449, 228.1451, **119.0499,**

								43.0504, 150.0315, 18.9471	229.1136, 107.0503, 246.0945, 139.1144
106	*P. lanceolata*	1.09	381.09511	1,21x10^{-14}	[M-H]-	C25H18O2S	Unclassified, sulfated	140.0932, 284.1295, 138.0811, 120.0623, 251.0021,	241.0031, **96.9582**, 243.0006, 261.0195, 130.0882
107	*P. lanceolata*	1.45	409.04766	3,02x10^{-06}	[M-H]-	C14H18O12S	Unclassified, sulfated	168.049, 312.0866,	241.0031, **96.9582**
108	*P. lanceolata*	1.83	439.05513	4,63x10^{-11}	[M-H]-	C15H20O13S	Unclassified, sulfated	198.0589, 342.0951,	241.0031, **96.9582**
109	*P. lanceolata*	2.03	373.11345	6,91x10^{-08}	[M-H]-	C17H18N4O6	Unclassified	206.072, 250.0623, 221.1056, 265.0892,	167.0372, 123.0437, 152.0125, 108.02,
110	*P. lanceolata*	2.8	373.11301	8,33x10^{-04}	[M-H]-	C16H22O10	Glycoside	176.0663, 164.0684, **180.0662**, **162.0484**, 220.0538	197.0441, 209.0445, 193.0407, 211.0557, 153.0559
111	*P. lanceolata*	3.13	433.14145	4,03x10^{-09}	[M-H]-	C18H26O12	Unclassified, Aromatic acid	236.0889, 251.1112, 295.1032,	197.0441, 182.0228, **138.0290**,
112	*P. lanceolata*	3.19	563.2283	6,34x10^{-01}	[M-H]-	C29H40O9S	Unclassified, sulfated, glycosylated	450.2041, 356.0899, 358.1094, 374.0974, 388.2034, 440.1885, 449.1997,	**113.0227**, 207.1380, 205.1253, 189.1234, 175.0228, 123.0437, 197.0441,

								366.1900, 400.1185, 390.1721, 462.2080,	163.1143, 173.0545, 101.0230, 198.0488, 208.1377, 561.2070, […]-, **96.95820**,
113	*P. lanceolata*	3.37	319.14009	2,61x10^{+00}	[M-H-H2O]-, [M+K-H]-	C24H20NO	Unclassified	163.0602, 107.0802, 102.0949, 127.0366, 206.0884, 42.9853, 93.9886, 151.9486, 226.1042, 85.9998, 205.0779, 233.0633, 96.0730, 160.1037	212.0509, 156.0701, 217.038, 192.106, 113.0543, 276.1546, 225.1366, 167.1936, 93.0325, 233.133, 114.0531, 86.071, 223.0602, 159.0343, 56.7092,
114	*P. lanceolata*	3.42	401.14426	2,20x10^{-20}	[M-H]-	C18H26O10	Polyphenole, Hydroxycinnamic acid	208.0963, 267.1050, 252.0821, 223.1152,	**193.0514**, 134.0357, 149.0596, **178.0264**,
115	*P. lanceolata*	3.45	431.12924	5,90x10^{-05}	[M-H]-	C18H24O12	Polyphenol, Hydroxy-cinamic acid, glycosylated	306.0961, 210.0398, 206.0395, 238.0668, 228.0484,	125.0235, 221.0823, 225.0840, **193.0514**, 203.0696, **113.0227**
116	*P. lanceolata*	3.49	613.21169	9,77x10^{-07}	[M-H]-	C23H38N2O17	Terpene, glycosylated	286.0763, 432.1611, 285.0889, 420.1549, 283.1095, 414.2023,	327.1156, 181.0567, 193.0514, 330.0960, 199.0149, 139.0371,

								474.1705, 284.0949, 402.1762, 486.1702, 397.2122, 415.1350, 348.3331	329.1183, 211.0377, 127.0393, 215.9960, […]-, **101.0230**, **456.1391**
117	*P. lanceolata*	3.53	963.27139	1,49x10^{-21}	[M-H]-	C44H52O24	Unclassified, Aromatic acid	482.1475, 682.2155, 481.1454, 681.2131, 724.2269,	481.1281, 281.0653, 282.0650, 239.0554, **137.0231**
118	*P. lanceolata*	3.54	544.13066	7,60x10^{-03}	[M-H]-	C19H29O18	Phenylpropa-noid	313.9847, 227.2967, 267.0734, 340.1032, 388.4210, 92.0767, 398.6884, 469.6728, 476.3490, 123.0123, 224.9926, 359.0406, 430.8272, 448.3846, 182.9811, 193.0199, 336.9861, 332.9850, 335.092,	230.1503, 316.8281, 277.0525, 204.0206, 452.0478, 155.7037, 145.4338, 319.1341, 421.1125, 185.0938, 74.4519, 67.7800, 95.7401, 207.1380, 361.1472, 351.1057, 113.3052, 209.0290, **193.0541**
119	*P. lanceolata*	3.65	459.15046	1,07x10^{-08}	[M-H]-	C25H22N3O6	Polyphenole, Hydroxy-cinnamic acid	186.0320, 166.0478, 214.0391, 250.1043, 198.0716, 63.1931,	208.0390, 273.1158, 293.1044, 245.1178, 209.0445,

								266.1291, 280.0749, 292.0852, […]-, 350.4543, 331.6202, 295.1032, 231.0468	261.0683, 395.9545, 193.0197, **179.0713**, 167.0718, 115.0827, […]-, 127.5274, **164.0452**,
120	*P. lanceolata*	3.66	533.12805	2,49x10^{-25}	[M-H]-	C32H22O8	Phenylpropa-noid	252.0539, 294.0667, 396.1114, 354.0959, 362.1182,	281.0653, 239.0554, **137.0231**, **179.0338**, 171.0102
121	*P. lanceolata*	3.67	669.10185	6,53x10^{-16}	[M-H]-	C28H46O18	Flavonoid, glycosylated	386.1610, 446.1635, **180.0581**, 384.1752, 354.1929, 404.1564, 532.2356, 448.1810, 460.2142, 462.2401,	283.1019, 223.0978, 489.2019, 285.0761, **315.0592**, 265.1088, 137.0231, 221.0823, 209.0445, **175.038**0
122	*P. lanceolata*	3.68	261.0437	8,50x10^{-03}	[M-H]-	C10H14O6S	Unclassified, sulfated	164.0885	**96.9582**, 261.0397,
123	*P. lanceolata*	3.77	525.16087	9,27x10^{-06}	[M-H]-	C24H30O13	Phenylpropa-noid	244.0894, 328.1141, 388.1335, 286.1062, 243.0908,	281.0653, 197.0441, **137.0231**, 239.0554, 282.0650, **341.0821, 179.0338**

124	*P. lanceolata*	3.88	399.16576	$6,34x10^{-01}$	[M-H]-	C21H24N2O6	Unclassified	194.0329, 303.8632, 190.1183, 121.0062, 186.0979, 205.0395, 179.0262, 107.0355, 180.0822, 64.0058, 188.0424, 327.3932, 59.7085, 172.0128	205.1253, 95.2950, 209.0445, 213.0493, 278.1513, 194.1225, 220.1339, 292.1146, 219.0665, 335.1512, 211.1110, 71.7649, 227.1440, 339.4497, 334.9702, 261.4741
125	*P. lanceolata*	3.92	565.22467	$1,92x10^{-07}$	[M-H]-	C27H36NO12	Unclassified	342.1249, 300.1165, 386.1154, 344.1074,	223.0978, 265.1088, 179.1067, 221.1235,
126	*P. lanceolata*	4	415.13734	$2,04x10^{-02}$	[M-H]-	C22H26NO7	Unclassified		
127	*P. lanceolata*	4.03	931.28229	$1,39x10^{-17}$	[M-H]-	C44H52O22	Iridoglycosid e	466.1498, 646.2148, 465.1496, 628.2046, 645.2119,	**465.1391**, 285.0761, 303.0864, **137.0231**
128	*P. lanceolata*	4.04	667.15112	$7,06x10^{-06}$	[M-H]-	C36H28O13	Iridoglycosid e	382.0677, 466.1498, 202.0042, 464.1505, 530.1196, 381.0654, 364.0579,	285.0761, 200.9925, **465.1391**, 202.9945, **137.0231**, 303.0864,

129	*P. lanceolata*	4.05	501.11652	$1,36x10^{-16}$	[M-H]-	C17H42O2S7	Unclassified, sulfated, glycosylated	216.0324, 364.0757, 365.0964, 260.0257, 352.0039, 380.0752, 318.0482, 293.0724, 305.0745, 263.0319, 215.0254,	285.0761, 137.0231, 136.0156, 241.0726, 149.0956, 121.0294, 183.0544, 208.0269, 196.0337, 238.0780, **113.0227, 956.9582**
130	*P. lanceolata*	4.11	611.22769	$9,56x10^{-04}$	[M-H]-	C26H44O16	Phenylpropa-noid	360.1238, 404.1125, 359.1208,	251.1261, 207.1380, 252.1254, **223.0602**
131	*P. lanceolata*	4.12	431.19138	$2,00x10^{-05}$	[M-H]-	C18H30N3O9	Unclassified	226.0637, 225.0653,	205.1253
132	*P. lanceolata*	4.16	495.13374	$1,64x10^{-16}$	[M-H]-	C13H36O13S3	Unclassified, sulfated, glycosylated	328.094, 374.0974, 180.0441, 358.1094, 329.1000, 344.1192, 373.0954, 360.0966, 256.0780, 290.0670, 382.1066, 343.1195, 372.0847, 346.0628, 342.1113, 286.0763,	167.0372, 121.0294, 315.0853, 137.0231, 166.0260, 151.0105, 122.0301, 135.0267, 239.0477, 205.0538, **113.0227**, 152.0125, […]-, **96.9582**

								199.0729, 283.0925,	
133	*P. lanceolata*	4.16	495.13374	$1,64x10^{-16}$	[M-H]-	C25H24N2O9	Unclassified	328.1047, 374.1104, 180.0441, 344.1303, 372.0958, 238.0668, 329.1173, 254.1281, 316.1153, 358.1094	167.0372, 121.0294, 315.0853, 151.0105, 123.0437, 257.0800, 166.0260, 241.0031, 179.0338, 122.0410
134	*P. lanceolata*	4.16	755.19918	$8,50x10^{-03}$	[M-H]-	C36H52O15S	Unclassified, sulfated	466.2613, 412.1719, 568.1836, 411.1702, 550.1708, 658.3337, 574.2259,	289.0396, 343.1210, 187.1123, 344.1169, 205.1253, **96.95820**, 181.0567,
135	*P. lanceolata*	4.22	725.28149	$1,16x10^{-10}$	[M-H]-	C32H46N4O15	Unclassified, glycosylated	412.1719, 520.1635, 476.1718, 538.1718, 436.2461, 574.2259, **180.0662**, 478.1574, 496.1614, 411.1702,	313.1029, 205.1253, 249.1120, 187.1123, 289.0396, 151.0569, 545.2193, 247.1304, 229.1228, 314.1236, **179.0561**
136	*P. lanceolata*	4.25	332.05639	$6,34x10^{-01}$	[M-H-H]-2	C32H26O16	Unclassified	58.0105, 130.8984, 174.9605, 76.0664, 117.9909,	274.0412, 201.1614, 157.0894,

								183.0018, 190.7880, 59.0133, 85.8280, 167.9738, 234.5058, 158.3924, 188.7560, 51.4247	255.9908, 149.0596, 214.0614, 141.2637, 273.0481, 246.2293, 164.0803, 97.5515
137	*P. lanceolata*	4.31	987.47169	7,68x10^{-08}	[M-H]-	C52H76O18	Glycoside	766.4291, 808.4418, 826.4512, 765.4254,	221.0671, **179.0561**, **161.0450,** 222.0683
138	*P. lanceolata*	4.34	403.1541	3,44x10^{-11}	[M-H]-	C18H28O10	Glycoside	180.0260, 179.0262, **162.0484**, 206.0395,	223.1343, 224.1387, 241.1095, 197.1206
139	*P. lanceolata*	4.3	611.19774	2,35x10^{-04}	[M-H]-	C28H36O15	Phenylpropa-noid	388.1335, 416.1286, 403.1583, 386.1154, 401.1443, 387.1343,	**223.0602,** 195.0663, 208.0390, 225.0840, 210.0557, 224.0616,
140	*P. lanceolata*	4.42	591.22857	7,53x10^{-09}	[M-H]-	C27H45O8PS2	Glycoside, sulfated, phosphoryla-ted	386.1029, 342.1249, 404.1125, 362.0969, 385.0982, 218.1025, 372.0847, 430.0969, 300.1067, 384.1622, 390.0991,	205.1253, 249.1120, 187.1123, 229.1228, 373.1294, 219.1366, **161.1346,** 291.1183, 207.0657, 201.1311, **196.0337**, **113.0227**, **96.9582**

								388.0924, 332.1020	
141	*P. lanceolata*	4.42	641.20718	8,50x10^{-03}	[M-H]-	C40H34O6S	Unclassified	418.1446, 432.1298, 433.1690, 434.1413, 417.1445, 424.0966,	223.0602, 209.0729, 208.0390, 207.0657, 224.0616, 217.1076,
142	*P. lanceolata*	4.5	395.16622	5,17x10^{-04}	[M-H]-	C17H33O8P	Unclassified, phosphorylated	236.2041, 203.1180, 339.388, 134.9786, 208.0811, 160.0734, 176.8273, 287.1648, 180.1185, 177.1379, 106.0145, 132.0464, 283.6564, 238.1911, 268.1105, 272.1598, 27.7607, 60.0774,	**158.9942**, 192.0732, 55.8039, 260.2117, 187.1123, 108.0200, 235.1195, 218.3653, 289.1785, 263.1309, 218.0588, 215.0682, 111.5362, 123.0437, 157.0084, 127.0783, 367.4328,
143	*P. lanceolata*	4.5	689.13778	8,85x10^{-01}	[M-H]-	C24H34O23	Phenylpropa-noid	466.0796, 481.1047, 465.0809, 448.1388,	**223.0602**, 208.0390, 224.0616, 241.0031,
144	*P. lanceolata*	4.55	595.20091	2,02x10^{-20}	[M-H]-	C28H36O14	Phenylpropa-noid, glycosylated	386.1154, 372.1388, 388.1335, 401.1443, 195.0756,	209.0729, **223.0602**, 207.0657, 194.0562, 400.1157,

								**180.0581**, 387.1602, 371.1294, 403.1583, 210.1032, 385.1166,	415.1369, 208.0390, 224.0616, 192.0411, **385.0927**, **205.0538,** **179.0338**
145	*P. lanceolata*	4.57	447.12876	7,53x10^{-09}	[M-H]-	C22H24O10	Flavonoid, glycosylated	**162.0484**, 268.0944, 161.0488, 161.9374, 296.0894, 206.0395, 265.0892, 163.0839, 258.0909, 192.0655, **180.0581**	285.0761, **179.0338**, 285.1942, 151.0379, 241.0880, 182.0439, **284.0352**, 189.0402, 255.0564,
146	*P. lanceolata*	4.57	609.18212	1,36x10^{-16}	[M-H]-	C28H34O15	Phenylpropa-noid	386.1258, 401.1443, 385.1262,	**223.0602**, 208.0390, 224.0616, **205.0538**
147	*P. lanceolata*	4.59	543.13373	4,12x10^{-01}	[M-H]-	C30H24O10	Phenylpropa-noid	258.0523, 406.0995, 240.0381, 407.1069, 257.0479,	285.0761, **137.0231**, 303.0864, 136.0156, **119.0499**
148	*P. lanceolata*	4.59	575.13916	3,84x10^{-06}	[M-H]-	C22H28N2O16	Unclassified, Aromatic acid	290.0670, 438.1202,	285.0761, **137.0231**,
149	*P. lanceolata*	4.6	507.15065	1,23x10^{-12}	[M-H]-	C24H28O12	Unclassified, Aromatic acid	222.0671, 370.1182, 371.1294,	285.0761, **137.0231**, 136.0156,

							266.0538, 386.1154, 221.0659,	241.0880, 121.0294,
150	*P. lanceolata*	4.63	341.12297	7,53x10^{-09}	[M-H]-	C16H22O8	Phenylpropa-noid, glycosylated	**162.0484**, 206.0316,	**179.0713**, 135.0797,
151	*P. lanceolata*	4.69	537.1457	3,23x10^{-05}	[M-H]-	C17H30O19	Flavonoid, glycosylated	400.1051, 414.0853, 392.0994, 416.1069, 370.0907, 390.0823, 344.0671, 340.0726, 332.0010, 238.0668, 318.0482, 469.9658, 294.9488, 322.1009, 332.0615,	137.0231, 123.0437, **145.0283**, 121.0294, 167.0372, 147.0460, 193.0656, 197.0607, 205.1253, **299.0585**, […]-, 80.9676, 138.0290, **78.9915,** **163.0491,** […]-, **341.1454,** **175.038**
152	*P. lanceolata*	4.7	451.16717	1,36x10^{-13}	[M-H]-	C22H28O10	Unclassified		
153	*P. lanceolata*	4.72	235.02909	1,51x10^{-22}	[M-H]-	C8H12O6S	Unclassified, sulfated	138.0811	9**6.9582**
154	*P. lanceolata*	4.72	459.19136	8,18x10^{-04}	[M-H]-	C21H32O11	Flavonoid, glycosylated	240.0509, 228.0484, 258.0523, 252.0821,	219.1366, 231.1375, 201.1311, 207.1031,

								294.1010,<br>233.1670,<br>132.0464,<br>174.1434,<br>239.0519,<br>245.0620,<br>217.1044,<br>253.0089,<br>294.1892,	165.0905,<br>226.0282,<br>327.1514,<br>**285.0397**,<br>220.1339,<br>214.1185,<br>242.0731, […]-,<br>**113.0227**, […]-,<br>**301.0291**
155	*P. lanceolata*	4.74	715.24055	$1,45x10^{-14}$	[M-H]-	C32H44O18	Flavonoid	548.2101,<br>492.1495,<br>563.2347,<br>468.1505,<br>450.1416,<br>486.1621,<br>512.1427,<br>547.2085,	167.0372,<br>223.0978,<br>152.0125,<br>247.0965,<br>265.1088,<br>229.0857,<br>203.1052,<br>168.0371,<br>**289.0995**
156	*P. lanceolata*	4.77	645.18102	$9,77x10^{-07}$	[M-H]-	C31H34O15	Phenylpropa-<br>noid	448.1388,<br>406.1239,<br>360.1102,<br>434.1118,<br>346.1055,<br>508.1584,<br>338.1568,<br>304.0911,<br>447.1317,	197.0441,<br>239.0554,<br>285.0761,<br>211.0694,<br>299.0744,<br>137.0231,<br>307.0299,<br>**341.0821**,<br>198.0488,
157	*P. lanceolata*	4.77	825.4243	$8,00x10^{-06}$	[M-H]-	C42H66O16	Glycoside	368.0952,<br>712.4032,<br>664.3807,<br>121.6460,<br>724.4035,<br>367.0945,<br>682.3956,	457.3456,<br>**113.0227**,<br>**161.0450**,<br>179.0561,<br>**101.0230**,<br>458.3335,<br>143.0330,

								706.39326, 666.39488, 694.39740, 604.3544	119.0333, 159.0343, 341.0821, **89.0284**
158	*P. lanceolata*	4.83	487.18138	$1,96x10^{-24}$	[M-H]-	C22H32O12	Glycoside	374.1533, 372.1754,	**113.0227**, 115.0035, **161.0450**
159	*P. lanceolata*	4.84	585.16223	$1,47x10^{-16}$	[M-H]-	C29H30O13	Iridoglycoside	300.0815, 448.1310, 346.0976, 304.0911, 406.1239, 299.0726, **120.0207**, 344.0671, 449.1422, 303.0869,	285.0761, 137.0231, 239.0554, 281.0653, **179.0338**, **465.1391**, 241.0880, 136.0156, 282.0650,
160	*P. lanceolata*	4.84	701.23201	$8,74x10^{-05}$	[M-H]-	C28H46O18S	Glycoside, sulfated	480.1687, 592.2047, 180.0822, 548.2163, 224.0596, 386.1610, 432.0326, 540.1900, 479.1146, 492.1834, 498.1636, 564.2050, 588.2029, 522.1348,	221.0671, 109.0263, 108.0200, 521.1496, 153.0242, 477.1843, 315.0592, 152.0125, 269.1975, **161.0450**, 222.1187, 209.0445, **137.0331**, 203.0696,

								502.1369, 404.1458,	**113.0338**, […]-, **101.023, 96.9582**
161	*P. lanceolata*	4.96	429.17621	5,32x10^{-10}	[M-H]-	C23H28NO7	Unclassified	208.0963, 226.1042, 224.0503, 207.0946,	221.0823, 203.0696, 205.1253,
162	*P. lanceolata*	5.01	411.20153	8,27x10^{-08}	[M-H]-	C28H28O3	Glycoside	**180.0581**, 174.0813, 218.0609, 288.1159, 204.2085, 290.1331, 211.2030, 265.3599, 179.0720, 145.1187, 89.0485, 203.1585, 188.5481, 144.1323, 214.1195, 86.0736, 154.0411, 64.0247,	231.1375, 237.1120, 193.1251, 123.0811, 206.9934, 121.0645, 200.0007, 145.8358, 232.1373, 266.0738, 322.1630, 208.0390, 222.6476, 267.0680, 197.0799, 325.1251, 257.1503, 347.1655, **113.1028**
163	*P. lanceolata*	5.01	495.06676	6,66x10^{-06}	[M-H]-	C15H30NO17	Unclassified	272.0884, 386.0780, 402.1088, 179.0542, 274.0569, 272.1354, 326.0743,	223.0602, 109.0651, 93.0325, 316.0904, 221.0823, 222.9975, 169.0636, 203.1439,

								291.9968, 242.0114, 262.0051, 288.0575, 374.1104, 377.7153, 447.1951, 246.0678, 220.0538	207.0831, 253.1273, 233.1330, 121.0294, 117.4256, 47.9458, 249.0706, 241.0031
164	*P. lanceolata*	5.05	387.12623	5,90x10^{-05}	[M-H]-	C12H14N13O3	Phenylpropa-noid, glycosylated	178.085, 163.0602, 177.071, **162.0484**, 15.0215, 221.0659,	209.0445, 224.0616, 210.0557, 225.0840, 372.1154, 166.0574, **223.0602**
165	*P. lanceolata*	5.08	489.19669	1,64x10^{-16}	[M-H]-	C27H30N4O3S	Phenylpropa-noid, glycosylated	376.1727, 254.1491, 316.1577, 374.1924, 338.0836,	**113.0227**, 235.0498, 173.0545, 115.0035, 151.1110, **191.0570, 101.0230, 161.0450**
166	*P. lanceolata*	5.19	661.29873	8,74x10^{-05}	[M-H]-	C31H50O15	Jasmonate conjugate	208.0598, 370.1106, 452.2622, 207.0535, 376.2285, 350.2251,	**291.1991**, 209.0445, 285.0761, 311.0794, 436.2371, 239.0554, 269.0481, 447.1288, **113.0227**, 309.2160,

								273.1882, 205.0864, **179.0561**
167	*P. lanceolata*	5.24	433.14949	3,02x10^{-06}	[M-H]-	C22H26O9	Glycoside	226.0869, 225.1122, 224.0707, 210.0920, 239.0958, 242.1122, 241.1100,	**207.0657**, 208.0390, 209.0729, 223.0602, 194.0562, 191.0361, 192.0411,
168	*P. lanceolata*	5.24	515.24891	7,37x10^{-10}	[M-H]-	C25H40O11	Flavonoid, glycosylated	238.0668, 164.0256, 237.0610, 182.0381, 256.0780, 402.2242, 378.1885, 226.0321,	277.1788, 351.2203, 333.2146, 259.1747, 513.2601, **113.0227**, 137.0588, 289.2157, 150.0691, 126.0156, […]-, **301.0291, 161.0450**
169	*P. lanceolata*	5.31	497.27506	8,50x10^{-03}	[M-H]-	C26H42O9	Polyphenole, Hydroxycinnamicacid	345.2560, **180.0662**, 360.4007, 377.2505, 308.2621, 336.3652, 235.1936, 281.2246, 362.2288, 272.2292, 314.4139, 89.1710, 241.0656	152.0125, 317.2055, 136.8697, 120.0167, 189.0120, 160.9046, **135.0432**, […]-, **175.0371**, 389.1669, 330.0960, 304.0708, 234.0175

170	*P. lanceolata*	5.37	417.16027	8,68x10^{-02}	[M-H]-	C19H30O10	unclassified, glycosylated	**180.0662**, 256.078, 212.0921, 179.0542, 304.1529	237.1120, 161.0966, 205.0864, 238.1105, **113.0227**,
171	*P. lanceolata*	5.57	357.15623	7,60x10^{-03}	[M-H]-	C12H26N2O10	Unclassified	61.9693, 174.1599, 156.1637, 125.0251, 120.0623, 185.0488, 75.1796, 156.0563, 104.032, 31.2981, 31.0489, 132.1735, 303.8424, 61.1324	295.1835, 182.9941, 200.9925, 232.1373, 237.0799, 172.1104, 281.9806, 201.1082, 253.1273, 325.8621, 326.1111, 224.984, 53.3178, 296.0315
172	*P. lanceolata*	5.59	469.24233	8,68x10^{-02}	[M-H]-	C24H38O9	Unclassified	222.1078, 266.0916,	247.1304, 203.1439,
173	*P. lanceolata*	5.63	519.20732	8,68x10^{-02}	[M-H]-, [M+HCOOH-H]-	C20H32N3O10	Unclassified	226.0637, 270.0602, 338.1194, 272.0738, 269.0565, 225.0653,	247.1304, 203.1439, 135.0797, 201.1311, 248.1310,
174	*P. lanceolata*	5.79	485.2021	8,85x10^{-01}	[M-H]-	C23H34O11	Unclassified	244.2044, 255.0548, 382.4200, 230.1984, 298.1505, 198.3316, 362.1566, 213.2209,	241.0031, 230.1503, 255.0144, 187.0679, 102.7854, 484.1997, 286.8736, 123.0437,

								377.6380, 205.0561, 283.1821, 223.1980, 267.1246, 297.1251, 317.2331, 364.1725	271.9867, 280.1451, 107.5641, 202.0248, 121.0294, 262.0039, 218.0779, 188.0813, 167.9754, 284.9143, 254.0189, 119.0499, 148.0485, 218.1987,
175	*P. lanceolata*	5.88	401.16258	$1,76x10^{-02}$	[M-H]-	C13H28N3O11	Unclassified	248.0848, 285.0889, 117.9715, 135.9825, 192.0511, 258.0909, 232.0752, 134.9786, 288.1027, 250.1043, 133.1276, 192.1804, 292.1586,	153.0751, 116.0722, 283.1922, 265.1845, 209.1166, 143.0712, 169.0861, 266.1760, 113.0543, 151.0569, 268.0361, 208.9816, 109.0098,
176	*P. lanceolata*	5.88	499.23635	$6,66x10^{-06}$	[M+HCOOH-H]-, [M-H]-	C24H38O8	Jasmonate conjugate	208.0522, 226.0637, 278.0821, 207.0535, 45.9943, 270.0801, 299.3244, 320.2154, 44.2115, 362.1717,	**291.1991**, 273.1882, 221.1555, 453.2537, 229.1727, 199.9259, **179.0338**, 455.0388,

								172.1443, 390.1881, 312.1465, 205.5912, 235.6828, 292.2795, 404.8488,	137.0846, 327.1156, 187.1123, 109.0651, 293.6591, 263.5676, 206.9708, 214.0227, 249.1865
177	*P. lanceolata*	6.07	389.21706	8,33x10^{-04}	[M-H]-	C17H32N3O7	Unclassified	45.1791, 223.1476, 188.1586, 110.1478, 202.1343, 239.1771, 90.0886, 149.1389, 147.0897, 178.2899, 304.9272, 158.1076	344.0284, 166.0574, 201.0552, 187.0679, 279.0583, 150.0288, 299.1152, 240.0632, 242.1178, 210.9177, 84.2804, 231.1065
178	*P. lanceolata*	6.08	443.19255	8,16x10^{-05}	[M-H]-	C21H32O10	Glycoside	256.078, 224.0596, **180.0662**, 255.0792, 258.1009	187.1123, 219.1366, 263.1309, 188.1093, 185.0938, **113.0227, 101.0230**
179	*P. pratensis*	4.11	621.10908	1,06x10^{-04}	[M-H]-	C27H26O17	Flavonoid, glycosylated	352.0665, 508.0875, 386.0557, 351.0645, 424.0709, 494.0733, 298.0519,	269.0481, **113.0227**, 235.0498, 270.0456, 197.0441, 127.0393, **285.0397**,

								336.0793,<br>398.0523,<br>270.0602,<br>412.0165,<br>399.0113,<br>443.6389,<br>446.0849,<br>384.0645,<br>353.9442,<br>421.0113,<br>433.0829,	323.0545,<br>223.0602,<br>351.0488,<br>209.0966,<br>222.0879,<br>**175.0228**,<br>177.4714,<br>267.1647,<br>237.0533,<br>188.0203,<br>**163.0359**	[…]-,
180	*P. pratensis*	4.23	651.12143	1,31x10^{-04}	[M-H]-	C28H28O18	Flavonoid	352.0665,<br>300.0667,<br>454.0776,<br>458.0846,<br>367.0945,<br>538.1011,<br>366.0783,<br>249.9423,<br>351.0645,<br>537.0940, 449.097,<br>536.1202,<br>362.0736,<br>318.0797,	**299.0585**,<br>351.0488,<br>197.0441,<br>193.0407,<br>**284.0352**,<br>**113.0227**,<br>**285.0397**,<br>401.1807,<br>**300.0661**,<br>202.0248,<br>115.0035,<br>289.0396,<br>333.0454,	
181	*R. acris*	3	477.16081	1,79x10^{-07}	[M-H]-,<br>[M+Cl]-,<br>[M+HC<br>OOH-<br>H]-	C22H26NO8	Unclassified	182.0952,<br>157.0641,<br>302.1690, 35.0362,<br>223.1152,<br>260.0689,<br>306.1315,<br>168.0490,<br>285.4405,<br>191.5267,<br>202.0911,<br>294.0928,	249.0551,<br>274.0830,<br>128.9830,<br>396.1143,<br>208.0390,<br>125.0235,<br>171.0768,<br>263.1035,<br>239.6238,<br>145.7075,<br>229.0568,	

								238.0668, 83.2776, 153.0899, 190.1519, 240.0670, 106.6210	137.0588, 347.8809, 193.0845, 241.0031, 191.0772
182	*R. acris*	3.27	577.21217	3,00x10^{+00}	[M-H]-	C28H36NO12	Unclassified	370.1106, 371.1181, 369.1042,	207.1031, 206.1004, 208.1073,
183	*R. acris*	3.37	559.17941	1,60x10^{-02}	[M-H]-	C25H36O14	Unclassified	369.1042, 368.0952, 326.0844,	190.0953, 191.1063, 233.1143,
184	*R. acris*	3.63	405.20243	6,10x10^{-02}	[M-H]-	C19H34O9	Unclassified, glycosylated	**162.0484**, **180.0581**, 161.0488,	243.1604, 225.1550, 244.1767, **113.0227,** 179.0542
185	*R. acris*	3.83	437.23277	3,97x10^{-07}	[M-H]-	C20H38O10	Unclassified	196.0805, 264.1700, 233.1051, 316.1577, 219.1051, 211.0677, 169.1731, 244.1499, 176.1273,	241.1420, 173.0545, 204.1264, 121.0645, 218.1309, 226.1580, 268.0693, 193.0845, 261.1088,
186	*R. acris*	3.84	420.09682	3,46x10^{+00}	[M-H]-	C19H19NO10	Unclassified	250.0716, 224.0503,	170.0267, 196.0491,
187	*R. acris*	3.85	567.25966	1,70x10^{-07}	[M-H]-	C25H44O14	Phenylpropa-noid, glycosylated	340.0923, 334.1868, 344.1436, 178.0364, 346.1957,	227.1640, 233.0644, 223.0978, 389.2190, 221.0671,

								406.2136, 342.1388, 302.1480, 454.2374, 344.1303, 177.0338, 388.1428, 376.1961, 442.2289, 420.2961, 466.2313	**161.0450**, 225.1095, 265.1088, 565.2573, **113.0227**, 223.1343, 390.2307, 179.1067, **191.0570**, 125.0235, 146.9501, **101.0230**
188	*R. acris*	4.19	471.18079	5,12x10^{-19}	[M-H]-	C22H33O9P	Unclassified, phosphorylated	67.9759, 208.0522, 248.0447, 210.0718, 166.0318, 230.0456, 232.1181, 66.9768, 164.0493, 224.0830, 247.0408, 205.0779, 216.1620, 270.1247, 252.0235	403.1997, 263.1309, 223.1343, 261.1088, 305.1371, 241.1420, 239.0554, 307.1193, 224.1387, 247.0965, 266.1090, **255.0144**, 201.0552, 219.1527, 263.1941
189	*R. acris*	4.42	381.12368	3,06x10^{-05}	[M-H]-	C19H26O9	Glycoside	206.0395, 262.1084, 205.0395, **162.0484**	191.1063, 135.0432, 192.1060
190	*R. acris*	4.47	405.17841	2,18x10^{+00}	[M-H]-	C18H30O10	unclassified, glycosylated	**180.0581**, 224.0503,	225.1095, 181.1174,

								182.0381, 179.0542,	223.1343, 226.1165,
191	*R. acris*	4.68	541.19507	2,45x10^{+01}	[M-H]-	C22H38O13S	Unclassified, sulfated	444.2385, 77.9504, **79.9615**, 239.9993, 242.0196, 76.9472,	**96.9582**, 463.2533, 461.2334, 301.2047, 299.1736, 464.2307,
192	*R. acris*	4.78	427.19573	6,23x10^{-05}	[M-H]-	C20H30NO9	Unclassified	206.1083, 224.1234, 224.0503, 266.0538,	221.0823, 203.0696, 203.1439, 161.1346,
193	*R. acris*	4.85	429.15844	5,30x10^{+01}	[M-H]-	C18H28N3O9	Unclassified	208.0963, 226.1042, 224.0503, 207.0946,	221.0823, 203.0696, 205.1253,
194	*R. acris*	4.96	427.14879	2,18x10^{+00}	[M-H]-	C20H30NO9	Unclassified	224.0395, 266.0538, 223.04, 206.1083, 222.0578,	203.1439, 161.1346, 221.0823, 205.1253,
195	*R. acris*	5.07	499.23821	1,21x10^{-01}	[M-H]-	C25H40O8S	Unclassified, sulfated, glycosylated	386.2082, 290.2499, 443.3236, 174.3693, 338.1807, 269.2135, 442.0312, 208.0811, 156.0563, 140.0479, 315.1747, 40.0953, 261.1846, 295.1862,	**113.0227**, 208.9816, 55.9070, 324.8613, 161.0450, 230.0083, 57.1994, 291.1478, 359.1826, […]-, 209.0966, 112.9376, 353.1090, **96.9582**, **101.023**0

								180.1450, 353.1346,	
196	*R. acris*	5.23	381.14863	8,46x10^{-01}	[M-H]-	C16H30O8S	Unclassified, sulfated	284.2012, **79.9615**,	**96.9582**, 301.2047,
197	*R. acris*	5.24	269.00934	1,21x10^{-16}	[M-H]-	C17H4NO3	Unclassified		269.0070
198	*R. acris*	5.62	415.17489	6,40x10^{-02}	[M-H]-	C23H28O7	Unclassified	211.1051, 62.0006, 318.2258,	415.1766, 204.0788, 353.1760, 96.9582,
199	*R. acris*	6.16	219.05794	3,06x10^{-05}	[M-H]-	C15H10NO	Unclassified	62.0006, 46.0083, 43.9916, 59.0133, 60.0239, 44.9985, 71.9868, 87.9814, 60.9952, 111.0559, 74.0352, 42.9853, 85.0308, 18.011,	157.0667, 173.0545, 175.0808, 160.0535, 159.0343, 174.0717, 219.0665, 147.0827, 172.0551, 131.0854, 158.0715, 108.0200,
200	*R. acris*	6.76	355.11841	1,72x10^{+00}	[M-H]-	C20H20O6	Phenylpropa-noid	236.0595, 210.0920, 192.0877,	**119.0499**, **145.0283**, **163.0359**

Table A3: List of plant traits including abbreviations, unit and description.

Trait	Abbreviation	Unit	Description
Leaf dry matter content	LDMC	mg/g	Leaf dry mass per leaf fresh mass
Specific leaf area	SLA	m^2/kg	Leaf area per leaf dry mass
Leaf area ratio	LAR	cm^2/g	Leaf area per total dry mass
Root dry matter content	RDMC	mg/g	Root dry mass per root fresh mass
Root to shoot ratio	RSR	g/g	Root dry mass per aboveground dry mass
Root volume	RVol	cm^3	Root volume
Root mass per volume	RMV	g/cm^3	Root dry mass per scanned root volume
Root carbon content	RCC	%	Root carbon content
Root nitrogen content	RNC	%	Root nitrogen content
Root carbon to nitrogen ratio	RCNR	g/g	Root carbon to nitrogen ratio
Root phosphorus content	RPC	µmol/g	Root phosphorus content
Root potassium content	RKC	µmol/g	Root potassium content
Root magnesium content	RMgC	µmol/g	Root magnesium content
Root calcium content	RCaC	µmol/g	Root calcium content
Root dry mass	DM roots	g	Root dry mass
Leaf dry mass	DM leaves	g	Leaf dry mass
Aboveground dry mass	DM above	g	including shoots, leaves and flowers
Total dry mass	DM total	g	Dry mass of whole plant
